# The first known fossil *Uma*: ecological evolution and the origins of North American fringe-toed lizards

**DOI:** 10.1186/s12862-019-1501-5

**Published:** 2019-09-06

**Authors:** Simon G. Scarpetta

**Affiliations:** Department of Geological Sciences, Jackson School of Geosciences, The University of Texas at Austin, 2275 Speedway Stop C9000, Austin, TX 78712-1722 USA

**Keywords:** *Uma*, Fossils, Divergence times, Lizards, Apomorphies, Paleoecology

## Abstract

**Background:**

Fossil evidence suggests that extant North American lizard genera (north of Mexico) evolved during the Miocene. Although fossils of the clade Phrynosomatidae (spiny lizards and sand lizards) have been reported, there have been no previously described fossils of the fringe-toed sand lizards (*Uma*). In the extant biota, *Uma* inhabit arid deserts, and members of the western clade of *Uma* are restricted to sand dunes or other habitats containing fine-grained sand.

**Results:**

I describe the first known fossil of *Uma* and refer the fossil to the total clade of *Uma* with an apomorphy-based diagnosis. The fossil is a partial premaxilla that was found in the Miocene strata of the Dove Spring Formation of southern California, dating to 8.77 Ma. The paleoenvironment of the Dove Spring Formation was semiarid and contained ephemeral streams that facilitated deposition, and there is no evidence of sand dune deposits in the strata containing the locality from which the *Uma* fossil was found. Divergence time analyses of a concatenated molecular dataset with four fossil calibrations support a Neogene origin of the total clade of *Uma* and of the crown clade of *Uma*. Those analyses also estimated a Neogene divergence between *Uma scoparia* and the *Uma notata* complex. Multispecies coalescent analyses with one fossil calibration inferred a Paleogene origin for the total clade of *Uma* and a Pliocene or Pleistocene divergence between *Uma scoparia* and the *Uma notata* complex. The fossil and the total and crown clades of *Uma* precede the evolution of modern desert ecosystems in the southwestern United States and northern Mexico by millions of years.

**Conclusions:**

The total clade and the crown clade of *Uma* were not restricted to arid deserts throughout their evolutionary histories. I demonstrate that an apomorphy-based diagnosis can be used to identify fossils of isolated skeletal elements for at least one clade of phrynosomatid lizard, and suggest exercising caution when using environmental tolerances of extant taxa to hypothesize paleoecological reconstructions.

**Electronic supplementary material:**

The online version of this article (10.1186/s12862-019-1501-5) contains supplementary material, which is available to authorized users.

## Background

Fossils that are similar to skeletal elements of extant lizard genera appeared in continental North America (North of Mexico) during the Miocene [1, 2]. However, the referrals of those and other Cenozoic lizard fossils to extant genera and species were not supported with apomorphic diagnoses in the context of modern hypotheses of phylogenetic relationships [3–5]. Diagnosis of fossils using apomorphies eliminates biases or errors that result from reliance on phenetic similarity or modern biogeographic distributions of species as tools to support identifications of fossils [3], and a fossil must be assigned to a clade using apomorphies or via phylogenetic analysis to be usable as a node calibration for that clade in divergence time analyses [6]. Distinguishing plesiomorphy from apomorphy is challenging, particularly for clades lacking phylogenetic clarity and when mostly fragmentary and disarticulated fossils are available for study, as is often the case for lizards [3, 6]. Moreover, many existing apomorphies were described with respect to an articulated skull, may be difficult to interpret on disarticulated elements, or were originally intended to diagnose higher-level relationships [3]. Still, some researchers have succeeded in using apomorphies to identify Cenozoic lizard fossils at the genus and species level [7–18].

Phrynosomatidae (horned lizards, sand lizards, spiny lizards, and relatives) is a species-rich and widely-distributed clade of North American lizards [19]. The two primary subclades within Phrynosomatidae are Sceloporinae sensu Wiens [19] (*Petrosaurus*, *Uta*, *Sceloporus*, *Urosaurus*), and Phrynosomatinae sensu Wiens [19] (*Phrynosoma*, *Uma*, *Callisaurus*, *Cophosaurus*, *Holbrookia*). Some Cenozoic fossils were previously referred without an apomorphy-based diagnosis to the sand lizard clade, which is composed of *Callisaurus*, *Cophosaurus*, *Holbrookia*, and *Uma* [20–27], but there is no known fossil record of *Uma* [28]. *Uma* is a clade of North American fringe-toed lizards whose extant representatives inhabit deserts in the southwestern United States and northern Mexico [29–31]. Some species of *Uma* are restricted to sand dunes or other desert habitats that contain loose, fine-grained sand [31, 32]. Sand dunes are ephemeral and easily disrupted ecosystems, and several extant species of *Uma* are at considerable conservation risk [32, 33].

**Fig. 1:**
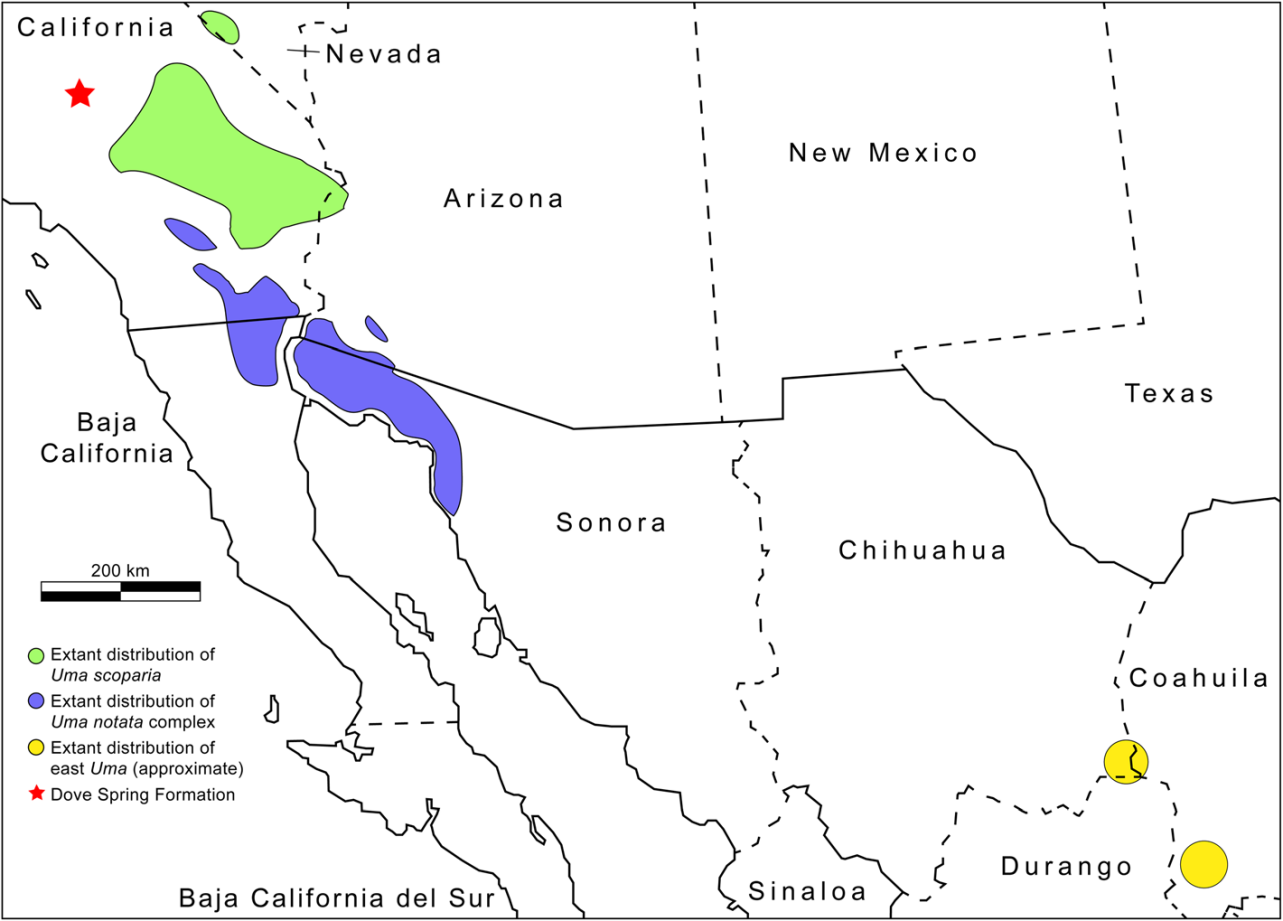
Current distribution of extant *Uma* clades and locality of the fossil (red star). The geographic distribution information was compiled from Stebbins [30], Gottscho et al. [32], iNaturalist [33], Williams et al. [34], Lemos Espinal and Smith [35], and Lemos Espinal and Smith [36]

**Fig. 2:**
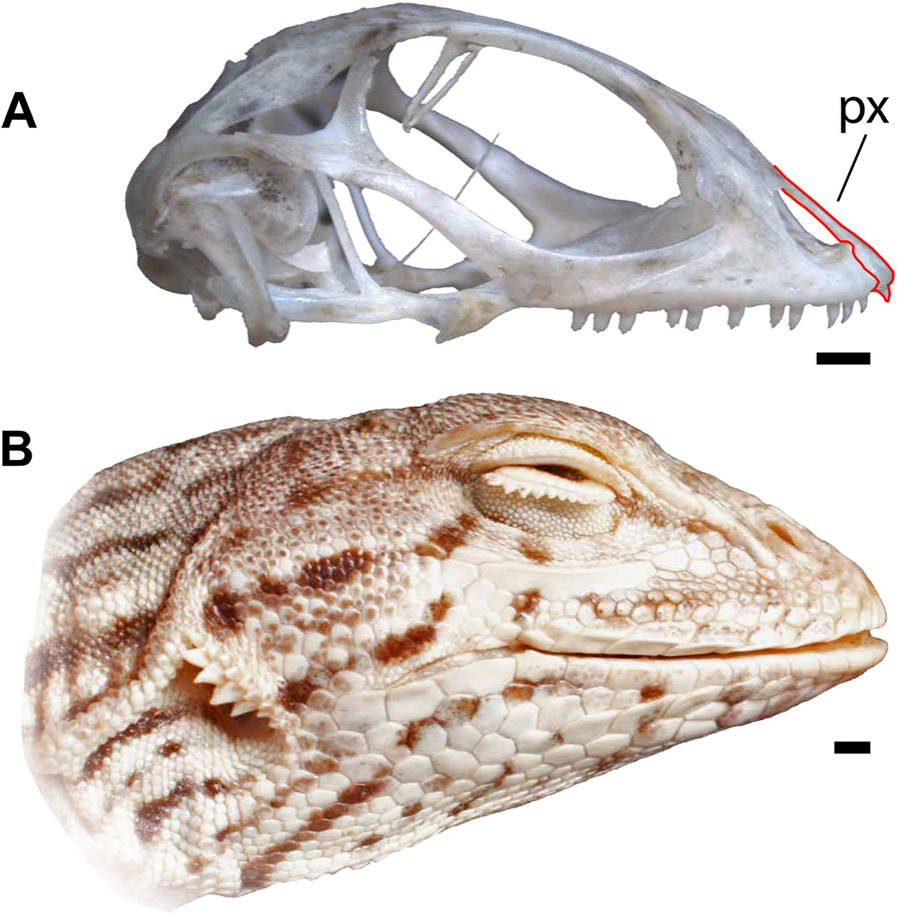
Right lateral views of *Uma notata*, scale bars = 1 mm. **a**. Modern skeletal specimen TxVP M-9950. The skull is shown excluding the mandible. The premaxilla (px) is outlined in red. **b**. Modern alcohol-preserved specimen TNHC 33314

*Uma* is split into two geographically distant clades, the western clade of *Uma* in the Sonoran and Mojave deserts of the southwestern United States and northwestern Mexico (*inornata*, *notata*, *rufopunctata*, *cowlesi*, an unnamed species, and *scoparia*) and the eastern clade of *Uma* (*exsul*, *paraphygas*) in the Chihuahuan Desert in north-central Mexico (Fig. 1) [33–37]. The western clade of *Uma* is divided into two subclades, the *Uma notata* complex (*inornata*, *notata*, *rufopunctata*, *cowlesi*, and an unnamed species) and *Uma scoparia*. Species in the western clade of *Uma* are restricted to habitats that contain loose, fine-grained sand (e.g., sand dunes, flats, washes, and riverbanks) [29–31]. Unlike the western *Uma*, *Uma exsul* and *Uma paraphygas* do not inhabit open dunes that are largely or completely devoid of vegetation and are known to frequent sandy soils containing hardened silt [38]. *Uma*, particularly the western clade, are morphologically and behaviorally distinctive lizards, possessing fringed-scaled toes, a shovel-like snout (Fig. 2), and an unusual sand swimming behavior [29–32].

Previous authors of phylogenetic analyses of *Uma* proposed Pleistocene divergence times between the *Uma notata* complex and *Uma scoparia* and suggested that Pleistocene glacial-interglacial cycles were responsible for diversification of those clades in conjunction with the development of modern sand-dune habitats [32, 33]. Those analyses were performed on population-level datasets and used multispecies coalescent methods. Time-calibrated analyses of concatenated datasets produced late Miocene [39] or early Pliocene [40] divergence times between *Uma scoparia* and the *Uma notata* complex, and a middle Miocene origin of crown *Uma*. Integrated analyses of mitochondrial DNA and geological data supported Miocene and Pliocene evolution of the lower Colorado River as the driver of vicariant speciation and diversification of *Uma scoparia* [41]. Miocene volcanism in the Sierra Madre Occidental was hypothesized to have resulted in the divergence of the eastern and western clades of *Uma* from each other [29].

I investigated the ecological and temporal origins of *Uma* using fossils, molecules, and geologic data. I describe the first known fossil of *Uma* and use an apomorphy-based diagnosis to refer that fossil to the total clade of *Uma*. I also provide new molecular divergence times for *Uma* and related lizards using both concatenated and coalescent phylogenetic methods and the newly described fossil and previously described fossils to calibrate nodes.

### Geologic and environmental setting

The fossil was recovered from the Miocene strata of the Dove Spring Formation, located in the Mojave Desert of south-central California (Fig. 1). The locality is ~ 200 km west of the nearest occurrence of extant *Uma*. The Dove Spring Formation is well-known for containing a large and diverse array of mammal fossils that exemplify one of the more complete faunal successions of the Clarendonian North American Land Mammal Age (NALMA), as well as fossils from the early Hemphillian NALMA [42].

Localities within the Dove Spring Formation contain fluvial, lacustrine, and volcanic sediments, and have good chronological control from several dated ashes and from paleomagnetic data [42]. The locality where the fossil *Uma* was found, LACM (Natural History Museum of Los Angeles County) 4702, contains strata composed of relatively coarse-grained alluvial fan deposits and paleosols with siliceous hardpans that indicate a semi-arid climate [42–44]. Microvertebrate fossils from the paleosols were interpreted as accretions of carnivore scat and collapsed burrows [44]. Robust grass macrofossils from the Dove Spring Formation c. 12 Ma suggest a relatively wet growing environment [45], and petrified wood referred to pines (*Pinus*), oaks (*Quercus*), cypress (*Cupressus*), and palms (*Palmoxylon*) were found in the Dove Spring Formation at localities near LACM 4702 [46]. Mammals reported from the locality LACM 4702 include shrews (*Alluvisorex*), canids (*Metalopex*), ringtails (*Bassariscus*), martens (*Martes*), squirrels (*Ammospermophilus*), and other rodents (*Cupidinimus*, *Perognathus*, *Paronychomys*, *Prosigmodon*) [42]. Other phrynosomatid lizards reported to occur in the Dove Spring Formation include *Sceloporus*, ? *Uta*, and *Callisaurus* [47]. The aggregate depositional environment, paleoflora, and paleofauna suggest a semi-arid but well-vegetated ecosystem containing ephemeral streams [42, 45].

### Temporal constraint

LACM 4702 is in the upper Dove Spring Formation. LACM 4702 and nearby localities are bracketed by radiometrically dated ashes at 9.7 ± 0.2 Ar/Ar [48] and at 8.5 ± 0.13 Ar/Ar [42, 47]. The mammalian fauna at LACM 4702 is characteristic of the Hemphillian NALMA, and the locality is in a magnetozone correlated with chron C4An, 9.11–8.77 Ma [42, 45, 49]. Based on the paleomagnetic data, I assigned LACM 4702 a minimum age of 8.77 Ma.

## Results

### Systematic paleontology

Squamata Oppel 1811 [50]

Iguania Cuvier 1817 [51]

Phrynosomatidae Fitzinger 1843 [52]

*Uma* Baird 1859 [53]

*Uma* sp. LACM 159892

### Description

LACM 159892 is a partial and fused premaxilla that preserves most of the main body of the element, some of the palatal process, and most of the teeth. The fossil also preserves the basal portion of the nasal process. Although some sediment partially obscures the posterior face of the specimen, the fossil was not prepared on the advice of the staff at both LACM and TxVP due to the small size and delicate nature of the specimen.

**Fig. 3:**
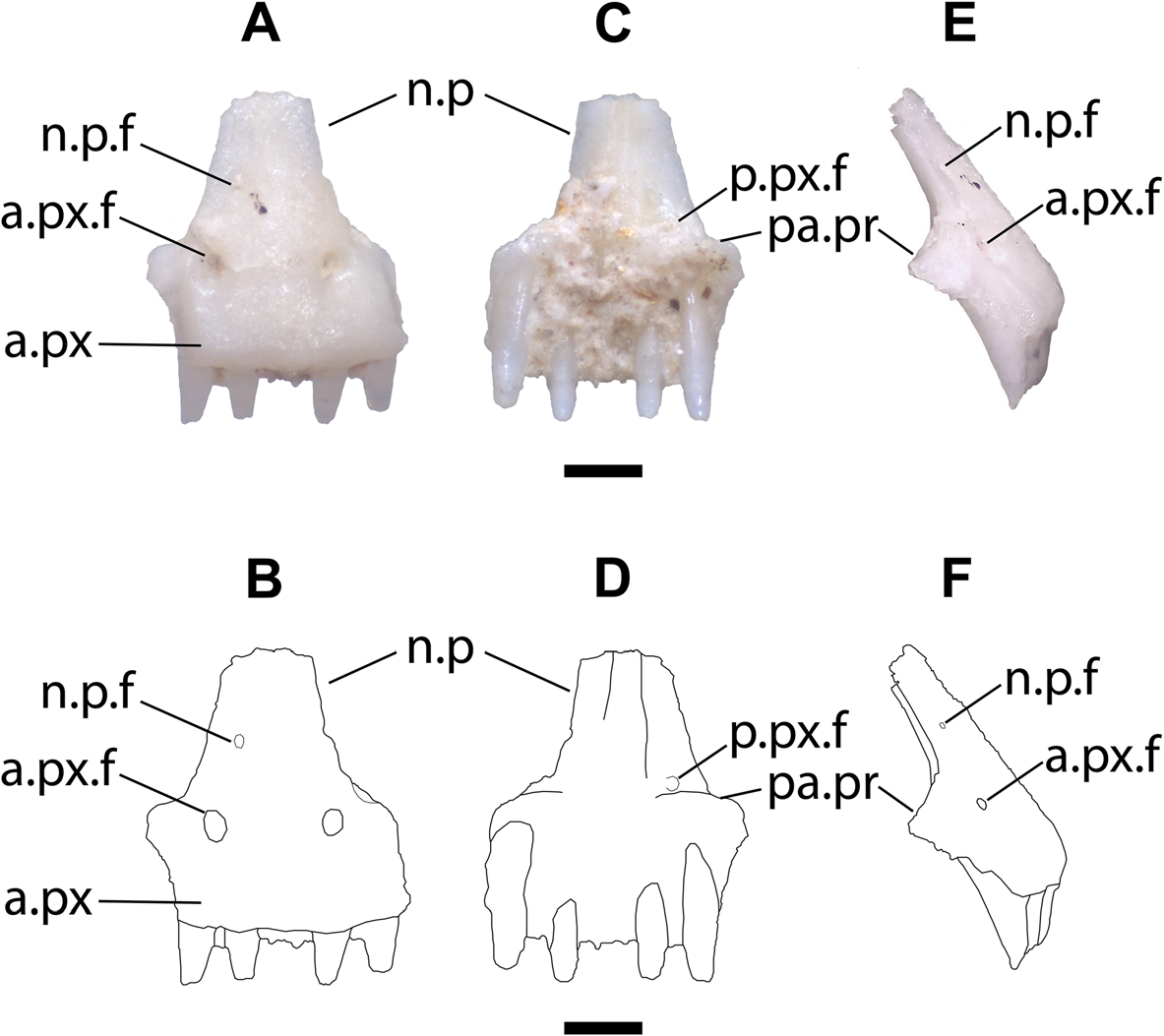
**a***. anterior* view of LACM 159892. Scale bar = 0.5 mm. **b.** Line drawing of LACM 159892 in anterior view. **c**. Posterior view of LACM 159892. **d**. Line drawing of LACM 159892 in posterior view. **e.** Right lateral view of LACM 159892. **f.** Line drawing of LACM 159892 in right lateral view. Anatomical abbreviations: a.px = anterior face of the premaxilla; a.px.f = anterior premaxillary foramen; n.p = nasal process; n.p.f = nasal process foramen; p.px.f = posterior premaxillary foramen; pa.pr = palatal process

The body of the premaxilla (the entire element excluding the nasal process) is rectangular when seen in anterior view and the nasal process is relatively wide, but is not as wide as the body of the premaxilla (Figs. 3a-d). The nasal process is directed posterodorsally. The nasal process and the anterior face of the premaxilla form a nearly flat surface (Fig. 3e-f). No dermal sculpturing is evident on the anterior surface of the element. A midline keel extends down the ventral face of the nasal process, nearly reaching the base of the process. There are two symmetrically placed anterior premaxillary foramina with corresponding posterior foramina that open just dorsal to the palatal portion of the element, and one anterior foramen on the nasal process. The fossil preserves five tooth positions with teeth that taper to a sharp point and are slightly recurved (Fig. 3). The right anterolateral side of the premaxilla is slightly broken, and if the broken portion were present, it probably would have contained an additional tooth position. The left anterolateral portion of the premaxilla preserves a facet for the maxilla.

### Diagnosis

I refer the specimen to Lepidosauria because the teeth are superficially attached to the jaw and to Squamata because the teeth are pleurodont and the premaxilla is a single element [54–56]. Among squamates, anterior premaxillary foramina are absent in Gekkota, Xantusiidae, Teiidae, Gymnopthalmidae, Scincidae, most other lacertiforms, and most anguimorphs [15]. The foramina are present in some taxa in Iguania, Cordylidae, and Gerrhosauridae, as well as in some amphisbaenians [15, 57]. Among Anguimorpha, anterior foramina are present in some members of Varanidae, Anguidae, and in most Xenosauridae [9, 15]. Almost all anguimorphs have at least seven tooth positions on the premaxilla [9, 58]. The exception is the extinct stem anniellid *Apodosauriscus minutus*, which has five tooth positions but has a triangular body of the premaxilla in anterior view and lacks anterior premaxillary foramina [59], distinguishing it from LACM 159892. LACM 159892 is not referable to Anguimorpha. Cordylids also have at least seven tooth positions, > 5 anterior foramina on the anterior face of the premaxilla, and a nasal process that is almost as wide as body of the premaxilla. Most gerrhosaurid specimens have > 7 tooth positions, but some specimens of *Zonosaurus* have five or six. However, the nasal process in gerrhosaurids curves posteriorly at its posterior end, and the anterior face of the premaxilla is rounded in lateral view. The premaxilla of Amphisbaenia differs from LACM 159892 by having a nasal process that is directed straight dorsally or curves anterodorsally relative to the body of the premaxilla [57]. Based on those morphological features, the fossil is referred to Iguania.

The premaxillae of members of crown Chamaeleonidae have a markedly narrow mediolateral dimension and either lack teeth or have vestigial teeth, distinguishing that clade from the fossil [60]. Most living Agamidae (excluding members of Uromastycinae, which have a hypertrophied central tooth position surrounded by denticles) have four or fewer pleurodont premaxillary teeth, except for *Japalura* and *Hydrosaurus*, which both can have up to five [60, 61]. *Japalura* and *Hyrdrosaurus* are distinguished from the fossil in that they do not have a rectangular premaxilla in anterior view, instead possessing a trapezoidal premaxilla in which the anterior margin is substantially wider than the palatal process [60]. Additionally, the ventral keel of the nasal process in *Hydrosaurus* is more pronounced than that of the fossil, and the nasal process of *Japalura* rapidly narrows dorsal to the base of the process. Although the fossil only preserves five tooth positions, it is probable that the broken anterolateral portion would have contained an additional position. The fossil is referred to Pleurodonta because it has five (and potentially six) teeth, a rectangular body of the premaxilla, and a narrow process that gradually narrows posteriorly.

Extant pleurodontan clades with anterior premaxillary foramina and that may have five or six tooth positions include Phrynosomatidae, Iguanidae, Hoplocercidae, Opluridae, and Leiosauridae [15, 18]. Hoplocercidae, Opluridae, and Leiosauridae form a clade, and Opluridae and Leiosauridae are sister taxa [62]. The anterior face of the nasal process is rounded in lateral view in Hoplocercinae [15]. In Leiosauridae and Opluridae, the anterior face of the premaxilla is mostly flat and the nasal process is directed dorsally, except for the posterior portion of the process that curves to face posteriorly. The nasal process of the oplurid *Chalarodon madagascariensis* is narrow relative to the body of the premaxilla, which has a curved anterior margin that gives the element a semicircular shape. The nasal process of *Oplurus cuvieri* is nearly as wide as the body of the premaxilla in anterior view, and the anterior margin of the premaxilla is wider than the palatal process. Dermal rugosities are present on the anterior face of the premaxilla in Leiosauridae and in some Opluridae. Iguanidae usually have multicuspid premaxillary teeth [63], while the teeth of LACM 159892 are unicuspid. Some specimens of *Ctenosaura* and *Sauromalus* (e.g., *Ctenosaura hemilopha* TxVP M-9258) and *Cyclura* have unicuspid premaxillary teeth [63]. The nasal process is relatively narrow and the anterior surface of the process is rounded in *Sauromalus* and *Cyclura* [18, 63]. The anterior face of the body of the premaxilla is rounded in *Ctenosaura*, and in *Ctenosaura hemilopha*, there is a groove on the dorsal surface of the nasal process that extends from the posterior end to the anterior end of the process. Although there are no known characters specific to the isolated premaxilla that explicitly diagnose Phrynosomatidae, LACM 159892 is referable to Phrynosomatidae through apomorphic process of elimination. In other words, assuming that the fossil is not a member of an unknown and extinct lizard clade, that it is not a stem member of another total clade within crown Iguania, and that it is not a stem member of Iguania, it must be a member of Phrynosomatidae.

**Fig. 4:**
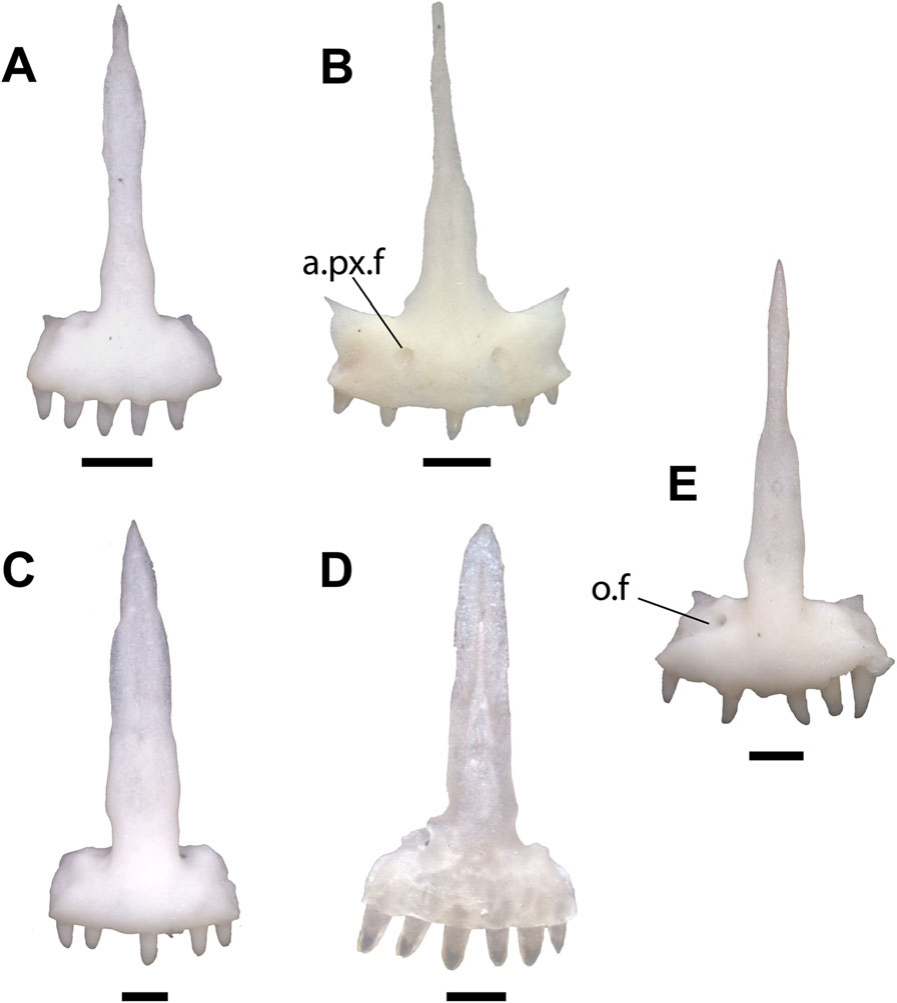
Premaxillae of sceloporine lizards in anterior view. Scale bars = 0.5 mm. **a**. *Urosaurus ornatus* TxVP M-14330. **b.** *Uta stansburiana* TxVP M-14331. **c**. *Sceloporus occidentalis* TxVP M-9817. **d**. *Sceloporus orcutti* TxVP M-12162. **e**. *Petrosaurus mearnsi* TxVP M-14910. Anatomical abbreviations: a.px.f = anterior premaxillary foramen; o.f = foramen on the dorsal surface of the body of the premaxilla

**Fig. 5:**
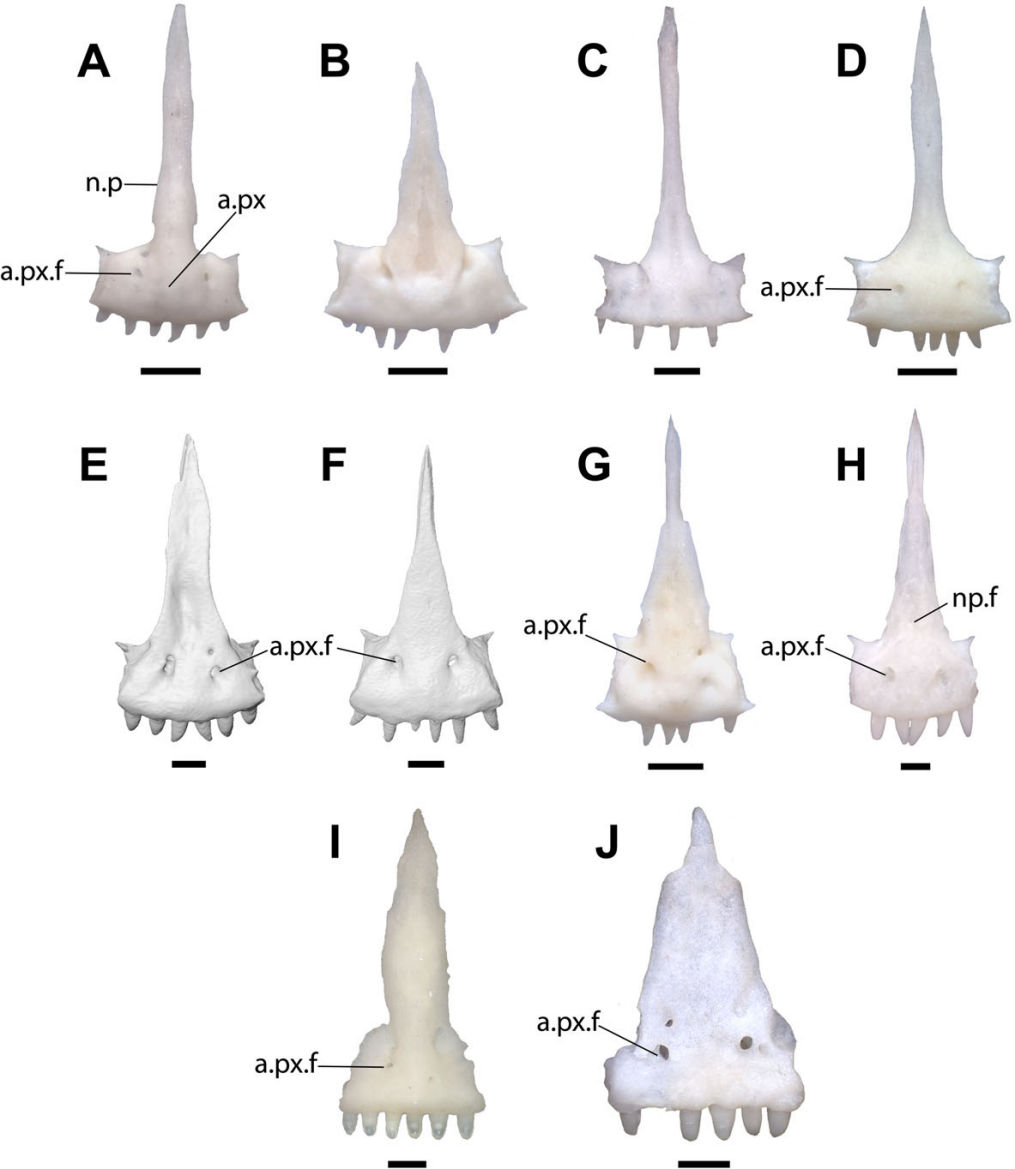
Premaxillae of phrynosomatine lizards in anterior view. Scale bars = 0.5 mm. **a**. *Cophosaurus texanus* TxVP 9219. **b**. *Holbrookia maculata* TxVP M-12128. **c**. *Holbrookia maculata* M-14322. **d**. *Callisaurus draconoides* TxVP M-8649. **e**. *Uma exsul* TNHC 30247 **f.** *Uma paraphygas* TNHC 30596. **g**. *Uma scoparia* TxVP M-12119. **h**. *Uma notata* TNHC 100800. **i*.*** *Phrynosoma platyrhinos* TxVP M-8954. **j**. *Phrynosoma cornutum* TxVP M-9621. Anatomical abbreviations: a.px = anterior face of the premaxilla; a.px.f = anterior premaxillary foramen; n.p = nasal process; n.p.f = nasal process foramen

Phrynosomatid taxa completely lacking anterior premaxillary foramina include *Sceloporus*, *Urosaurus*, and *Petrosaurus* (Fig. 4a, c, d, e). *Petrosaurus* (Fig. 4e), *Sceloporus*, *Cophosaurus*, and *Holbrookia* occasionally have a separate anterior foramen on the anterodorsal surface of the body of the premaxilla. Anterior premaxillary foramina are present in some specimens of *Uta stansburiana* (Fig. 4b), but the premaxilla of *Uta* is distinguished from the fossil by possessing a relatively narrow nasal process compared to the width of body of the premaxilla and a rounded anterior face of the premaxilla. Anterior foramina are also present in *Phrynosoma* (Fig. 5i, j), but the premaxilla of *Phrynosoma* is distinguished from other phrynosomatids in having a vertical and flat anterior face and a nasal process that is directed dorsally. Among phrynosomatids, only taxa in the sand-lizard clade have a premaxilla with anterior foramina, a flat anterior face, and a posterodorsally directed nasal process.

The premaxillary teeth of all sand-lizards have unicuspid crowns that taper to a point and are often slightly recurved, as in the fossil. The teeth of *Phrynosoma* are blunt and peg-like, although some taxa possess more robust teeth (Fig. 5i, j). All examined *Uma* had either four or six premaxillary tooth positions, while *Holbrookia* had five to eight tooth positions and *Callisaurus* and *Cophosaurus* had four to eight tooth positions.

**Fig. 6:**
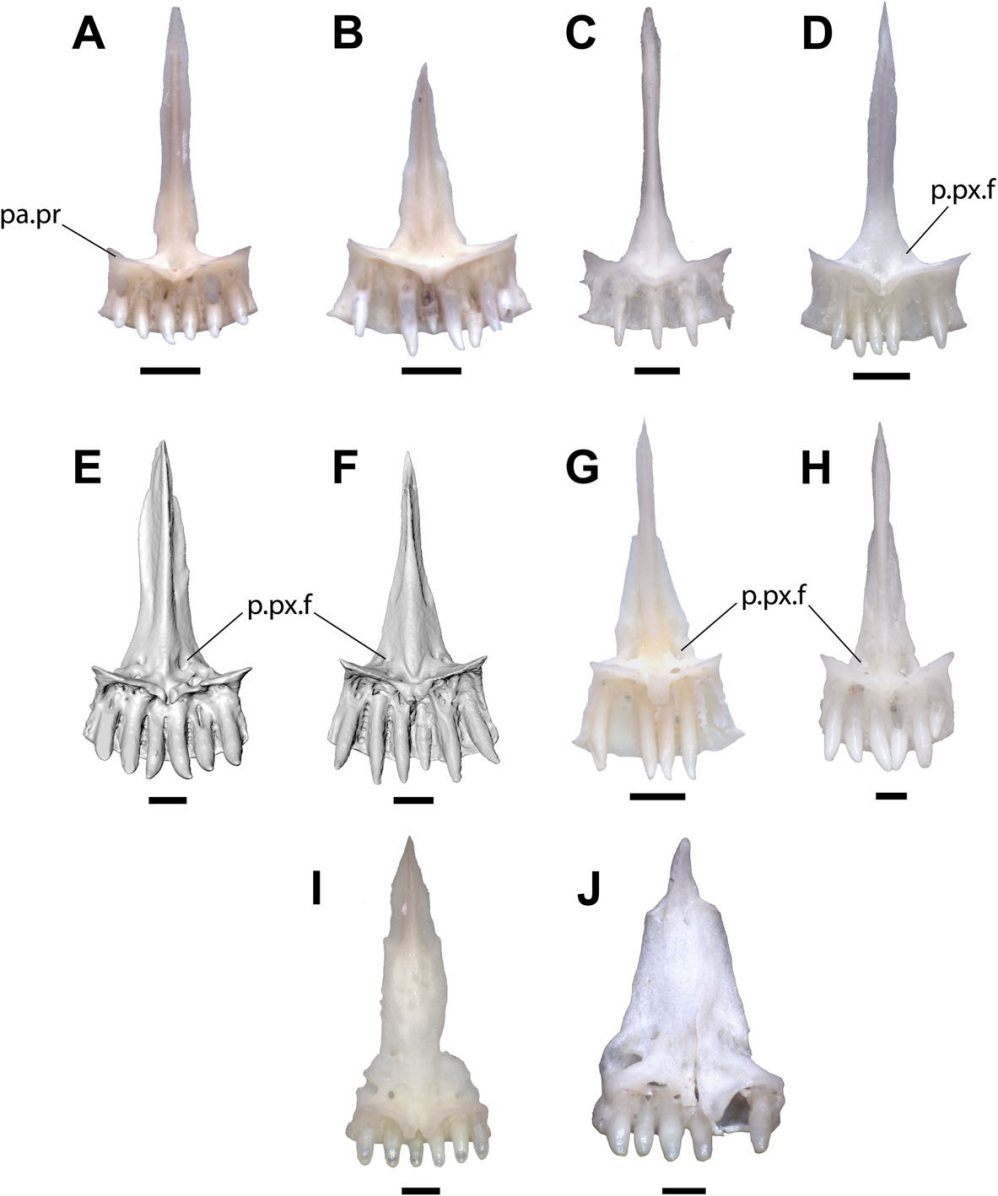
Premaxillae of phrynosomatine lizards in posterior view. Scale bars = 0.5 mm. **a**. *Cophosaurus texanus* TxVP 9219. **b**. *Holbrookia maculata* TxVP M-12128. **c**. *Holbrookia maculata* M-14322. **d**. *Callisaurus draconoides* TxVP M-8649. **e**. *Uma exsul* TNHC 30247 **f.** *Uma paraphygas* TNHC 30596. **g**. *Uma scoparia* TxVP M-12119. **h**. *Uma notata* TNHC 100800. **i*.*** *Phrynosoma platyrhinos* TxVP M-8954. **j**. *Phrynosoma cornutum* TxVP M-9621. Anatomical abbreviations: pa.pr = palatal process; p.px.f = posterior premaxillary foramen

Anterior foramina were previously noted in *Uma* and *Callisaurus*, but not in *Holbrookia* or *Cophosaurus* [15]. I observed anterior foramina in all specimens of *Uma* and most specimens of *Callisaurus* (Figs. 5d-h) and in some specimens of *Holbrookia* (e.g., TNHC 18387) and *Cophosaurus* (Fig. 5a). There are one to four foramina, which may be symmetrically arranged or haphazardly distributed. Two symmetrical foramina on the anterior face of the premaxilla are present in all examined specimens of *Uma inornata* (*n* = 3) and *Uma paraphygas* (*n* = 2), in three of four specimens *Uma exsul* and four of five specimens of *Uma notata*, and in some specimens of *Callisaurus* and *Holbrookia*. The posterior openings of the anterior foramina are usually bilateral and symmetrical (Fig. 6d-f). Like the fossil, extant *Uma* and *Callisaurus* may have anterior foramina on the nasal process, while other phrynosomatids do not.

The relative width of the nasal process was previously used to differentiate iguanian taxa [15, 16, 64]. A wide nasal process was previously observed in *Uma* compared to other sand-lizards, but was not used as a systematic character because of interspecific variation [28]. I also observed intra- and interspecific variation in the morphology and relative width of the nasal process in *Cophosaurus*, *Callisaurus*, and *Holbrookia*, but the morphology of the process is largely consistent among specimens of *Uma* that I examined. The nasal process in *Uma* and LACM 159892 is relatively wide at its base compared to other sand lizards. The base of the nasal process is considerably wider than the rest of the process in a few specimens of *Callisaurus* (Fig. 5d), but the process rapidly narrows dorsal to the base of the process.

The nasal process of *Uma* is shaped like an isosceles triangle, where the two long margins of equal length are oblique and gradually taper to a sharp point posteriorly (5E-5H). In *Uma scoparia* (Fig. 5g), *Uma exsul* TNHC 30248, and *Uma notata* TNHC 100800 (Fig. 5h), the nasal process abruptly narrows posteriorly (= reduction of the lateral crests of de Queiroz [28]). A comparable morphology is present in *Phrynosoma cornutum* (Fig. 5j) and in *Urosaurus ornatus* (Fig. 4a). In some specimens of *Uma exsul* the nasal process remains wider posteriorly (Fig. 5e). The distal portion of the nasal process of LACM 159892 is broken, but the preserved portion gradually narrows in width from the base to the preserved posterior margin, as in extant specimens of *Uma*. The nasal process of *Holbrookia* sometimes resembles that of *Uma*, but the body of the premaxilla is always much wider than the width of the nasal process in *Holbrookia* (Fig. 5b, c). Specimens of *Phrynosoma* also have wide nasal processes with roughly parallel lateral margins (Fig. 5i) or with lateral margins that taper towards the posterior end (Fig. 5j). However, the bases of the nasal processes in all *Phrynosoma* I examined are as wide or are nearly as wide as the body of the premaxilla. Within Phrynosomatinae, a wide, posterodorsally-directed nasal process that is not as wide at its base as the body of the premaxilla and is shaped like an isosceles triangle is apomorphic of *Uma*.

The body of the premaxilla of most sand-lizard specimens is rectangular when seen in anterior view. In *Uma*, particularly *Uma notata* and *Uma inornata*, the body of the premaxilla is not as wide as in other sand-lizards, and the premaxilla has a comparatively square shape (Fig. 5e, f, g, h). The shape of the premaxilla is unique to *Uma* among phrynosomatids and contributes to the perception of a wide nasal process, so those characters cannot be considered independent. The fossil has a relatively square-shaped premaxilla. LACM 159892 exhibits a derived suite of features present in all extant *Uma* but does not possess any apomorphies specific to any clade within the crown of *Uma*. I refer the fossil to the total clade of *Uma*.

The premaxilla of *Uma* is differentiated from that of all other squamates by possessing the following combination of characters: foramina on the anterior face of premaxilla, foramina may be present on the anterior face of the nasal process, anterior face of the premaxilla and nasal process form a flat anterior surface, nasal process is directed posterodorsally, nasal process relatively wide and shaped like an isosceles triangle, body of the premaxilla square in anterior view relative to other North American sand lizards (*Callisaurus*, *Cophosaurus*, and *Holbrookia*), teeth pointed and slightly recurved, and four to six tooth positions present. The entire diagnosis is summarized in Table 1 below.

**Table 1:** Summary of the systematic diagnosis of the fossil LACM 159892

Positive Evidence			
Morphological Feature	Hypothesis of character evolution	Alternative hypothesis	Reference
Teeth that are superficially attached to the jaw	Apomorphy of Lepidosauria	–	[54]
Pleurodont teeth	Apomorphy of Squamata	–	[55]
Fused postnatal premaxilla	Apomorphy of Squamata	–	[54, 56]
≥5 premaxillary tooth positions	Apomorphy of Pleurodonta within crown Iguania, independently acquired in *Hydrosaurus* and *Japalura*	–	[60, 61]
5–6 premaxillary tooth positions	Independently derived in Phrynosomatidae, Iguanidae, and Opluridae + Leiosauridae + Hoplocercidae	–	[15, 18]
Anterior premaxillary foramina (across Squamata)	Independently derived in Iguania, Anguimorpha, Cordylidae + Gerrhosauridae, Amphisbaenia	Independently derived in Iguania + Anguimorpha, Cordylidae + Gerrhosauridae, Amphisbaenia	[15, 18]
Anterior premaxillary foramina (within Phrynosomatidae)	Apomorphy of sand lizards within crown Phrynosomatidae, independently derived in *Uta*	Apomorphy of Phrynosomatidae, secondarily lost in *Sceloporus*, *Urosaurus*, and *Petrosaurus*	[15], this paper
Anterior face of the premaxilla and nasal process form a flat anterior face	Apomorphy of sand lizards within crown Phrynosomatidae	–	This paper
Rectangular body of premaxilla	Apomorphy of sand lizards within crown Phrynosomatidae	–	This paper
Posterodorsally directed nasal process	Apomorphy of sand lizards within crown Phrynosomatinae	–	This paper
Isoceles triangle-shaped nasal process	Independently acquired apomorphy of *Uma* and of *Holbrookia*	Apomorphy of sand lizards, lost in *Callisaurus* and *Cophosaurus*	This paper
Anterior foramen on the nasal process	Independently acquired apomorphy of *Uma* and of *Callisaurus*	Apomorphy of sand lizards, lost in *Holbrookia* and *Cophosaurus*	This paper
Relatively wide nasal process (dependent on shape of premaxilla)	Present only in *Uma* within sand lizard clade	–	[28]
Relatively square body of premaxilla	Apomorphy of *Uma*	–	This paper
Negative Evidence			
5–6 premaxillary tooth positions	≥7 premaxillary tooth positions is an apomorphy of Anguimorpha and of Cordylidae + Gerrhosauridae (see text for exceptions)	–	[9, 58]
Posterodorsally directed nasal process	Apomorphy of sand lizards, absent in Amphisbaenia	–	[57]
Anterior face of the premaxilla and nasal process form a flat anterior surface	Apomorphy of sand lizards, absent in Iguanidae, Hoplocercidae, Cordylidae + Gerrhosauridae	–	[15]
Rectangular body of premaxilla	Apomorphy of sand lizards, absent in Opluridae + Leiosauridae	–	This paper
Unicuspid teeth	The presence of multicuspid premaxillary teeth is an apomorphy of Iguanidae (see text for exceptions)	–	[63]

The first section lists positive evidence for membership of the fossil in each clade, and the second section lists negative evidence for the inclusion of the fossil in a given clade. The reference column does not necessarily indicate that the character was considered an apomorphy by the author(s) who described the morphological feature

### Additional material

**Fig. 7:**
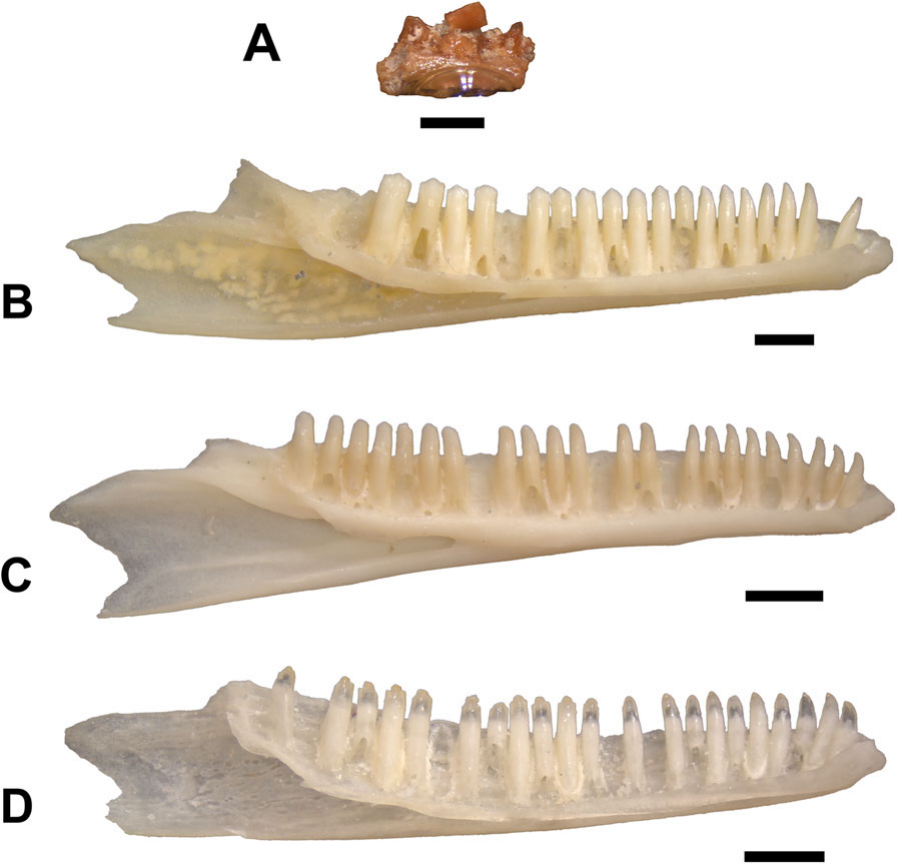
Dentaries of modern phrynosomatid lizard specimens and a fossil from the Dove Spring Formation in lingual view. Scale bars = 1 mm. **a.** LACM 159954. **b.** *Uma scoparia* TxVP M-12119. **c.** *Callisaurus draconoides* TxVP M-14320. **d.** *Sceloporus occidentalis* TxVP M-12176

I examined > 800 lizard fossil specimens from LACM. Approximately 350 of those specimens preserve morphologies comparable to that of extant phrynosomatid lizards, including weakly tricuspid teeth and a closed but unfused Meckel’s canal for part of the dentary. Most specimens are fragmented dentaries and maxillae, and almost all are not referable using apomorphies to a clade less inclusive than total clade Phrynosomatidae. None of those specimens are referable to *Uma* using apomorphies. Excluding *Phrynosoma* [7], the dentaries and maxillae of phrynosomatid lizards are not consistently diagnostic for clades that are currently ranked as genera. That said, there are at least four specimens that preserve tooth morphology consistent with that of extant species of *Uma*, although the specimens preserve no apomorphies of *Uma*. Those specimens are LACM 159790 (maxilla), LACM 159954 (dentary), LACM 159744 (dentary), LACM 159717 (dentary). The dentary teeth of *Uma* generally implant more ventrally on the dental shelf than those of other phrynosomatid lizards, and the dentary and maxillary teeth of the western clade of *Uma* are more robust than those of other sand lizards (Fig. 7). Additionally, specimens of the western clade of *Uma* (Fig. 7b) and the fossil LACM 159954 (Fig. 7a) have dentary teeth that are weakly tricuspid, but that have a blunt central cusp. The dentary and maxillary tooth morphology of *Uma* overlaps to some degree with *Sceloporus* and *Petrosaurus* and the crotaphytids *Crotaphytus* and *Gambelia*, so although I suggest a potential affinity of the above fossils with *Uma*, I do not refer those fossils to *Uma* for the time-being. All of those specimens were also found at the locality LACM 4702 except for LACM 159954, which was found at LACM locality 4697, situated lower in the Dove Spring Formation c. 12 Ma [42].

### Remarks

Some descriptions of phrynosomatid fossils evaluated the morphology of the dentary of *Uma* [1, 26, 65], one author evaluated the skeletal morphology of sand lizards including *Uma* [28], and one morphometric analysis of *Uma* was conducted previously [66]. Apomorphic characters were never identified on the premaxilla or any other skeletal element of *Uma* until now. Fragmentary fossil material of *Uma* is identifiable when elements containing sufficient phylogenetic information are discovered, and the same may be true of other clades within Phrynosomatidae.

### Non-clock phylogenetic analysis of concatenated dataset

I conducted a non-clock phylogenetic analysis of molecular data of Phrynosomatidae and other iguanian taxa to provide a topology for strict- and relaxed-clock analyses (see Methods). All nodes in the non-clock analysis were supported with a posterior probability of 1.0, and it is noteworthy that the interrelationships of the sand-lizard clade were resolved with strong support. *Callisaurus* and *Holbrookia* were placed as sister taxa and *Cophosaurus* was the sister to that clade. That result is corroborated by most recent analyses [39, 67, 68]. Some analyses of molecular data inferred a monophyletic earless-lizard clade of *Cophosaurus* and *Holbrookia* [19, 69–72], which was supported by analyses of morphological data [28, 73].

### Strict-clock analysis of concatenated dataset

I performed strict-clock analyses to estimate clock rates for the relaxed-clock analyses (see Methods). The median tree height (TH) was 0.2220 for the analysis of the entire dataset, 0.1096 for the analysis of the nuclear data, and 1.616 for the analysis of the mitochondrial data.

### Relaxed-clock analysis of concatenated dataset

I conducted relaxed-clock divergence time analyses in BEAST v1.10 [74] with two different models, a model with one mean clock rate for the entire dataset, and a model with two mean clock rates, one for the nuclear data and one for the mitochondrial data (hereafter referred to the one-rate and two-rate models). Those analyses included four node calibrations (see Methods). I also analyzed both models with all fossil calibrations except the sand lizard calibration to test the sensitivity of the divergence times to the calibration, and those analyses included three calibrations. Additionally, I conducted analyses for both models that included all fossil calibrations and in which the divergence of the western clade of *Uma* was constrained to the Pleistocene, and those analyses included a total of five calibrations.

**Fig. 8:**
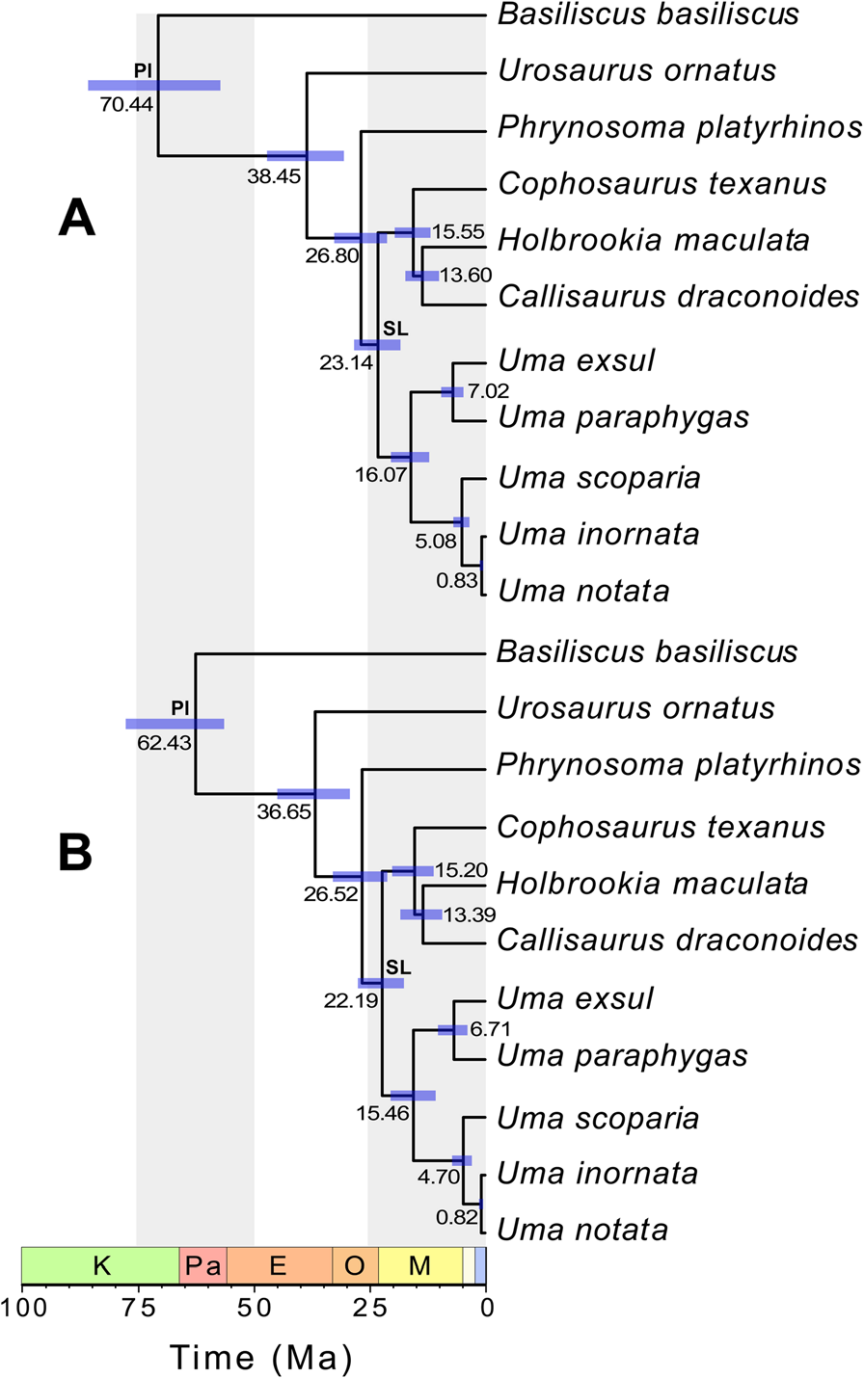
Time-calibrated phylogenies of pleurodontan lizards from analyses with all fossil calibrations included. The two-rate analysis is the analysis with the best support overall. Acrodont outgroups are excluded. Numbers at nodes are median divergence times and purple bars represent 95% HPD intervals. Letters above nodes indicate where fossil calibrations were applied; Pl = Pleurodonta, SL = sand lizard. The time scale is in millions of years and period abbreviations above the scale are as follows: K = Cretaceous; Pa = Paleocene; E = Eocene; O = Oligocene; M = Miocene. The light-yellow period is the Pliocene and the blue period is the Pleistocene. **a**. One-rate analysis. **b**. Two-rate analysis

Middle-Miocene ages were previously inferred for the crown node of *Uma*, including at 18.1 Ma [39], 15.9 Ma [40], and ~ 15 Ma [75]. Those estimates are supported by both sets of analyses here that did not include the Pleistocene calibration (Fig. 8; Table 2). Earlier authors proposed divergence times of 7.1 Ma [39], ~ 5 Ma [75], 4.5 Ma [40], or approximately 1 Ma [32, 33] between the *Uma notata* complex and *Uma scoparia*. My analyses without the Pleistocene calibration produced divergence times like those of Wiens et al. [40] and Van Dam and Matzke [75], supporting an early Pliocene split between the *Uma notata* complex and *Uma scoparia* (median age 4.68–5.13 Ma) with likely minimum and maximum ages in the late Pliocene and the late Miocene, respectively (Table 2, Fig. 8).

**Table 2:** Summary of divergence times from concatenated analyses for the sand lizard, *Uma*, western *Uma*, and eastern *Uma* crown clades

One-rate analyses				
Model	Sand lizard clade	*Uma*	Western *Uma*	Eastern *Uma*
All fossil calibrations	23.14 [18.31, 28.29]	16.07 [12.13, 20.36]	5.08 [3.47, 6.93]	7.02 [4.76, 9.54]
^a^No sand lizard calibration	23.56 [18.70, 29.25]	16.32 [12.29, 20.75]	5.13 [3.53, 7.01]	7.09 [4.84, 9.71]
All fossil calibrations + Pleistocene calibration	18.51 [11.98, 25.80]	12.34 [7.10, 18.39]	1.70 [0.95, 2.64]	5.39 [2.35, 9.07]
Two-rate analyses				
Model	Sand lizard clade	*Uma*	Western *Uma*	Eastern *Uma*
^**a**^ **All fossil calibrations**	**22.19 [17.51, 27.43]**	**15.46 [10.67, 20.38]**	**4.70 [2.86, 7.12]**	**6.71 [3.80, 10.16]**
No sand lizard calibration	22.53 [17.57, 27.99]	15.63 [10.74, 20.92]	4.68 [2.83, 7.20]	6.79 [3.79, 10.31]
All fossil calibrations + Pleistocene calibration	18.81 [14.10, 23.92]	12.66 [7.38, 18.38]	2.00 [1.33, 2.77]	5.41 [2.10, 9.62]

The two-rate analysis with all fossil calibrations (in bold) is the overall best supported analysis, based on the Bayes Factor analyses (see below). The best supported analysis for each clock rate model is denoted by a ^a^. Numbers outside of brackets are median divergence times and numbers in brackets indicate the 95% highest posterior density (HPD) intervals

I estimated median divergence times of 6.71–7.09 Ma for the eastern clade of *Uma* (divergence between *Uma exsul* and *Uma paraphygas*) in the two sets of analyses without the Pleistocene calibration, similar to other analyses [39, 40, 75]. The median age for the split between *Uma notata* and *Uma inornata* was 0.82–0.83 Ma, almost identical to some prior analyses [39, 40] but older than others [32, 33]. Divergence estimates for other nodes were comparable to those found by some researchers [39, 40], although the basal divergence within crown Phrynosomatidae and the divergence of *Uma* from the other sand lizards (Table 2, Fig. 8) were substantially younger than other estimates [67, 68].

Divergence time estimates in the analyses without the sand lizard calibration are slightly older (median divergence times < 0.5 Ma) than estimates from the analyses with all calibrations (Table 2). Divergence estimates in the analyses with the Pleistocene calibration were younger than those in both other analyses.

### Model comparisons of concatenated analyses

I conducted path-sampling and stepping-stone analyses [76, 77] in BEAST v1.10 to estimate marginal likelihoods to evaluate the support for each model. The Bayes Factor (BF) test statistic 2log_e_BF was used to evaluate the support for each model. A given model was interpreted as very strongly favored when 2log_e_BF > 10, strongly favored when 2log_e_BF > 6 and < 10, positive when 2log_e_BF > 2 and < 6, and not worth mentioning when 2log_e_BF < 2, modified from the recommendations of Kass and Raftery [78].

The two-rate models were very strongly favored over the one-rate models (Table 3). The analyses with only fossil calibrations were very strongly favored over those that also included the Pleistocene calibration for the western clade of *Uma* (Table 3) for both the one-rate and two-rate analyses, so an early Pliocene divergence is favored between *Uma scoparia* and the *Uma notata* complex. The two-rate model with all fossil calibrations was the best supported analysis overall.

**Table 3:** Marginal likelihood estimates from path-sampling and stepping-stone analyses and 2log_e_BF test statistics comparing evidence for different analyses

One-rate analyses				
Model	Path Sampling log_e_ (Marg. Lik.)	Stepping Stone log_e_ (Marg. Lik.)	Path Sampling 2log_e_BF	Stepping Stone 2log_e_BF
All fossil calibrations	−111,014.69	−111,014.42	3.24 (181.58)	3.58 (181.60)
^a^No sand lizard calibration	−111,013.07	− 111,012.63	(178.34)	(178.02)
All fossil calibrations + Pleistocene calibration	−111,023.13	− 111,022.44	20.12 (197.64)	19.62 (198.46)
Two-rate analyses				
Model	Path Sampling log_e_ (Marg. Lik.)	Stepping Stone log_e_ (Marg. Lik.)	Path Sampling 2log_e_BF	Stepping Stone 2log_e_BF
^a^**All fossil calibrations**	**−110,923.90**	**−110,923.62**	**–**	**–**
No sand lizard calibration	−110,924.78	− 110,924.17	1.76	1.10
All fossil calibrations + Pleistocene calibration	−110,932.31	−110,931.95	16.82	16.66

The two-rate analysis with all fossil calibrations (in bold) is the best supported analysis overall. The favored analysis for each clock rate model is denoted by a ^a^. The 2log_e_BF values are the favored model compared to the given model. For the one-rate analyses, the 2log_e_BF outside of parentheses are in comparison to the one-rate analysis without the sand lizard calibration, and 2log_e_BF in parentheses are in comparison to the overall favored analysis (two-rate analysis with all fossil calibrations)

Surprisingly, the analysis without the sand lizard fossil calibration was slightly favored over the analysis with the calibration for the one-rate analyses (2log_e_BF = 3.24–3.58). There was no positive evidence for the model with the sand lizard calibration over the model without the sand lizard calibration for the two-rate analyses (2log_e_BF < 2). In either case, including all appropriate calibrations is my preferred analytical strategy and the divergence times in the analyses without the sand lizard calibration were almost the same as those that included the calibration, so all discussions and figures of the concatenated analyses refer to the results of the one- and two-rate analyses with all fossil calibrations.

### Multispecies coalescent analysis

I performed multispecies coalescent analyses on a Sanger dataset of *Uma* that includes multiple individuals of each terminal taxon, and I added representatives of each of the other sand lizards (*Callisaurus*, *Cophosaurus*, *Holbrookia*) to allow for a node calibration. Multispecies coalescent analyses were conducted in BEAST 2.5 [79] using *BEAST.

**Fig. 9:**
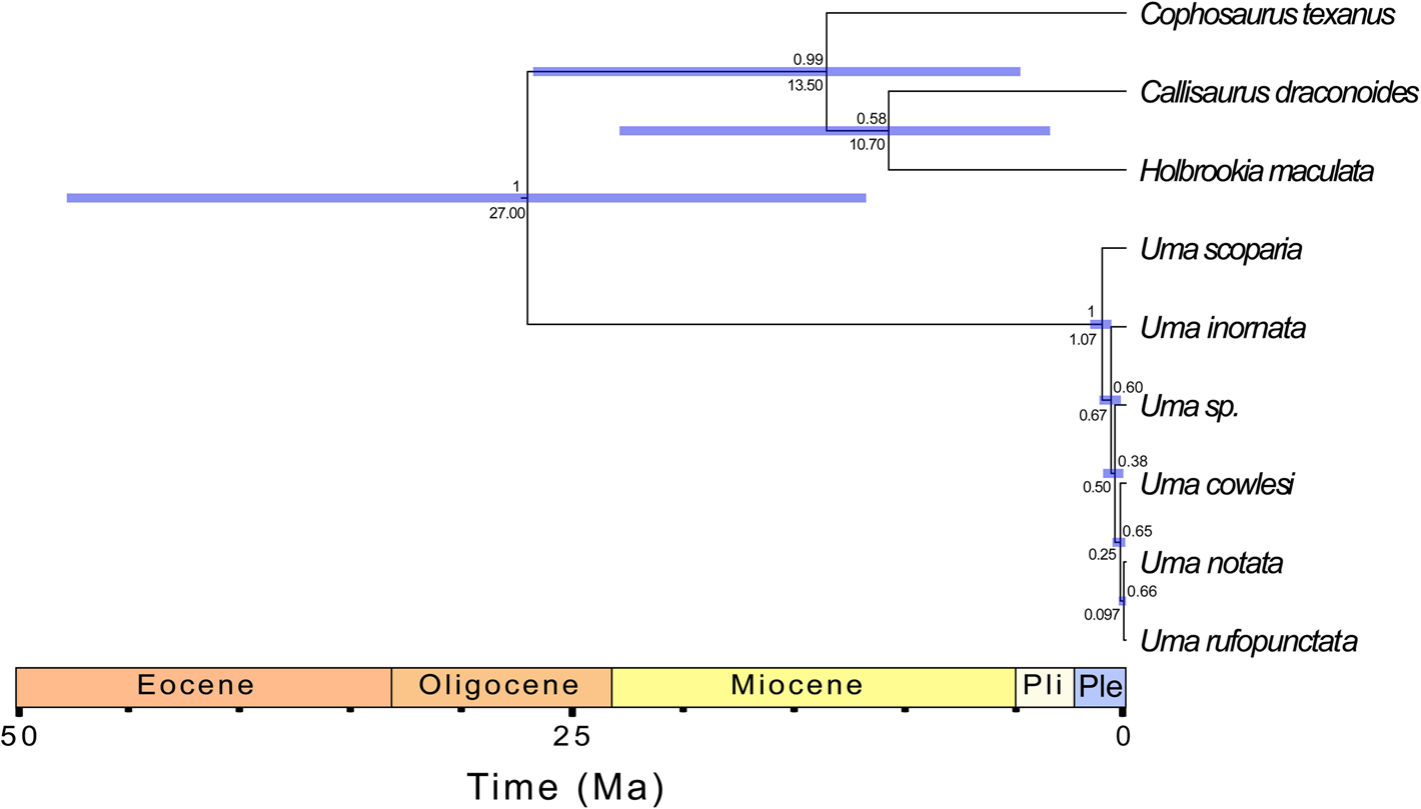
Best supported multispecies coalescent analysis with a single fossil calibration at the sand lizard node and a Pleistocene calibration at the western *Uma* node. Numbers below nodes are median divergence times, purple bars represent 95% HPD intervals for the estimated divergence times at each node, and numbers above nodes are the posterior probabilities for each node. Pli = Pliocene; Ple = Pleistocene

Interrelationships of the sand lizard genera were the same as those inferred in the concatenated analyses, although the sister taxon relationship between *Holbrookia maculata* and *Callisaurus draconoides* was estimated with low posterior probability (Fig. 9). As in a previous study that used Sanger data for species delimitation in *Uma*, the interrelationships of the *Uma notata* complex were estimated with low support [33].

I analyzed three different models: a model with only the sand lizard fossil calibration, a model with the sand lizard calibration and a Pliocene calibration for the western clade of *Uma*, and a model with the sand lizard calibration and a Pleistocene calibration for the western clade of *Uma* (see Methods). In the analysis with only the fossil calibration and the analysis that also included the Pliocene calibration, the split between *Uma scoparia* and the *Uma notata* complex was inferred to have occurred during the middle to late Pliocene (median age 3.79–2.72 Ma; Table 4). In those analyses, a middle Eocene (median 47.93–41.39 Ma) divergence was inferred between *Uma* and the other sand lizards, similar to or slightly older than previous analyses of concatenated phylogenomic datasets [67, 68]. Analysis of the model that included the sand lizard calibration and the Pleistocene calibration, however, produced substantially different divergence times. In that analysis, I found an early Pleistocene (median 1.07 Ma) divergence of *Uma scoparia* from the *Uma notata* complex, and a late Oligocene (median 27.00 Ma) split between *Uma* and the other sand lizards. A late Oligocene origin for the total clade *Uma* is similar to the concatenated analyses that did not include a Pleistocene calibration. For all three coalescent analyses, the 95% HPD intervals for the sand lizard crown node were not tightly constrained (Table 4, Fig. 9).

**Table 4:** Summary of divergence times from multispecies coalescent analyses for the sand lizard clade and the western clade of *Uma*

Model	Sand lizard clade	Western *Uma*
Sand lizard calibration only	41.39 [16.35, 75.62]	2.72 [0.66, 6.28]
Sand lizard calibration + Pliocene calibration	47.93 [22.40, 79.46]	3.79 [2.20, 5.77]
**Sand lizard calibration + Pleistocene calibration**	**27.00 [11.72, 47.78]**	**1.07 [0.65, 1.61]**

The analysis with the sand lizard calibration and the Pleistocene calibration for the crown node of the western clade of *Uma* (in bold) is the best supported analysis based on the Bayes Factor analyses (see below)

### Model comparisons of multispecies coalescent analyses

I assessed support for the different coalescent models by conducting stepping-stone analyses in BEAST 2.5 and comparing the resulting marginal likelihoods with the 2log_e_BF test statistic. Surprisingly, both models that included a calibration at the crown western *Uma* node were strongly or very strongly favored over the model that did not (2log_e_BF = 8.42–18.74), but the model with the Pleistocene calibration was very strongly favored over the model with the Pliocene calibration (2log_e_BF = 10.32; Table 5).

**Table 5:** Marginal likelihood estimates from stepping stone analyses and 2log_e_BF test statistics comparing evidence for different analyses

Model	Stepping Stone log_e_ (Marg. Lik.)	Stepping Stone 2log_e_BF
Sand lizard calibration only	− 5226.25	18.74
Sand lizard calibration + Pliocene calibration	− 5222.04	10.32
**Sand lizard calibration + Pleistocene calibration**	**− 5216.88**	**–**

The analysis with the sand lizard calibration and the Pleistocene calibration for the crown node of the western clade of *Uma* (in bold) is the best supported analysis

## Discussion

### Origin and ecological evolution of *Uma*

In the concatenated analyses, I estimated a middle Miocene origin for the crown clade of *Uma* with 95% HPD intervals ranging from the early Miocene to the late middle Miocene, and a middle Oligocene to middle Miocene divergence of *Uma* from the other sand lizards (Table 2). Given those ages, LACM 159892 is approximately 1–12 Ma younger than the age of crown *Uma* and 9–21 Ma younger than the age of the total clade *Uma*. The multispecies coalescent analysis with the best support inferred a median divergence time during the middle Oligocene between *Uma* and the other sand lizards (Table 4). The lowest known stratigraphic datum in the fossil record for any given taxon necessarily postdates the first historical appearance of that taxon in its evolutionary history [80], so in most cases the oldest known fossil of a clade will postdate molecular divergence times of that clade [81]. Older fossils of stem and crown *Uma* will almost certainly be discovered given more extensive sampling of Oligocene and Miocene sediments from the southwestern and western United States and northern Mexico.

There is no stratigraphic evidence of sand dunes in the Dove Spring Formation [42]. However, sand dunes are selectively and often incompletely preserved in the rock record, so the absence of compound cross-strata indicating the presence of a mature dune field [82] does not by itself eliminate the possibility that dunes may have existed. The coarse sediments in the stratum in which LACM 4702 was deposited as well as the paleofloral and -faunal assemblages of LACM 4702 and adjacent localities provide no circumstantial evidence of sand dunes or other habitats containing loose, fine-grained sand [42]. The lineage of *Uma* to which LACM 159892 belonged does not appear to have inhabited fine-grained sand. That early *Uma* were not specialized sand-dwellers was previously hypothesized by Norris [29], who recognized that it was improbable that *Uma* could have spread over such a wide range if the clade had arisen adaptively in response to the development of sand dune habitats. He instead suggested that early *Uma* occupied flood plains [29], a similar depositional environment to that of the Dove Spring Formation. *Uma exsul* and *Uma paraphygas* inhabit sandy desert environments but are less specialized for dwelling in loose sand than species of the western clade [38], which supports the hypothesis that the common ancestor of the western and eastern *Uma* clades and other early members of the *Uma* total clade were not sand dune or fine-grained sand specialists. Although the fossil LACM 159892 is not referable to the crown clade of *Uma*, it is noteworthy that the Dove Spring Formation is located close to the modern range of species in the western clade of *Uma*.

Neogene global cooling and aridification that precipitated the formation of modern arid ecosystems did not begin until the late Miocene [83]. While some semi-desert plants appeared in North America during the Miocene, desert vegetation communities like those currently present in the Mojave and Sonoran deserts did not appear until the late Pliocene or Pleistocene and the region did not attain its modern aridity until the Pleistocene [84–87], millions of years after both the divergence of *Uma* from the other sand lizards and the basal divergence of crown *Uma*. Similarly, extant desert vegetation communities of the Chihuahuan Desert did not exist until the Pliocene or Pleistocene [88, 89] and the area possibly did not attain its modern level of aridity until the Pleistocene [90]. The aggregate molecular, paleontological, and geological evidence suggests that early representatives of both the total clade and of the crown clade of *Uma* did not inhabit the desert environments to which extant species of *Uma* are restricted. The development of modern desert habitats does not appear to have played a role in the early evolution of *Uma*, and correspondingly, the modern ecological and environmental tolerances of extant *Uma* are not necessarily analogous to those of early stem or crown *Uma*.

The presence of *Uma* in habitats outside of the ecological constraints of the modern species has important consequences for researchers who use fossils to infer past environments. The environmental tolerances of the extant relatives of extinct organisms often are used to develop paleoecological reconstructions [91]. As discussed here and elsewhere [91, 92], species do not always inhabit the same environments in the present that they inhabited in the past. Extant lizard species, for example, can exhibit rapid phenotypic and ecological responses to environmental changes [93, 94]. Ecological niche models that assume static environmental tolerances through time or that the realized niche is the same as the fundamental niche produce biased predictions of mammal species distributions under different climate regimes [95]. The evolution of ecological tolerances through time must be considered when using fossils to reconstruct paleoenvironments [92], and potential errors in paleoecological interpretations based on the extant biota will be greatest when reconstructions are based on taxa with narrow environmental tolerances and relatively specialized extant ecologies, such as *Uma*.

### Origin of the western clade of *Uma*

I found a Pliocene divergence between *Uma scoparia* and the *Uma notata* complex in analyses of the concatenated dataset (Table 2). Bayesian hypothesis testing very strongly favored Pliocene divergence between *Uma scoparia* and the *Uma notata* complex over Pleistocene divergence. Those analyses temporally support the Neogene vicariance hypothesis, in which the development of the lower Colorado River and sequential marine inundations in the Colorado Desert region of the Sonoran Desert during the Miocene [96, 97] facilitated the divergence of the *Uma notata* complex from *Uma scoparia* [41]. Coalescent analyses estimated a middle-late Pliocene or a middle Pleistocene divergence between *Uma scoparia* and the *Uma notata* complex (Table 4), but the latter was very strongly favored. That result supports the hypothesis that Pleistocene glacial-interglacial cycles drove speciation within the *Uma notata* complex as well as the divergence of *Uma scoparia* [31, 32].

Given the results of the concatenated analyses, diversification of the western clade of *Uma* occurred during a period of global aridification, which was accompanied by the proliferation of grassland ecosystems across North America [83, 98]. Sand dunes in the extant range of *Uma notata* appeared as early as 4.3 Ma when silt from the Colorado River reached the Salton Trough [96]. *Uma* may not have inhabited sand dunes until around the time that *Uma scoparia* and the *Uma notata* complex split from each other, implying that the divergence of those clades was associated with the initial development of sand dunes during the early Pliocene instead of the development of modern dune formations during the Pleistocene. On the other hand, if the divergence between *Uma scoparia* and *Uma notata* occurred during the Pleistocene, as supported by the favored coalescent analysis, then the origin of the western clade of *Uma* was associated with the evolution of both modern sand dunes and modern deserts, and was moderated by recent glacial-interglacial cycles [32, 33]. More Neogene and Pleistocene fossils of *Uma* and additional phylogenomic data for *Uma*, particularly *Uma exsul* and *Uma paraphygas*, will shed further light on these hypotheses.

## Conclusions

The fossil record is the most direct source of evidence for reconstructing past ecosystems, but integrative approaches incorporating fossils, molecules, and geologic data are necessary to limit biases that adversely affect paleoecological and paleoenvironmental interpretations. Given the early history of the North American fringe-toed sand-lizards and echoing concerns previously raised by Behrensmeyer [99], I caution against paleoecological inferences based directly on the environmental tolerances of the extant relatives of extinct organisms. Paleoecology of past extinction events is often used to inform future conservation decisions, and the earth is currently undergoing a mass extinction [100–103]. Thus, it is increasingly important to develop and conduct paleoecological datasets and analyses, respectively, that do not rely on questionable assumptions with respect to fossil identification [3] and paleoecological interpretation ([92], this paper).

## Methods

### Collection and conservation of the fossil

LACM 159892 was collected by D. Whistler on May 19, 1981. The fossil is housed at the paleontological collections of the Natural History Museum of Los Angeles County (LACM).

### Selection of comparative sample

Osteological nomenclature follows Evans [60] unless otherwise noted. I compared the fossil to a variety of taxa by reviewing literature of squamate skeletal anatomy and by examining specimens. I examined disarticulated specimens when possible because the fossil is a single, disarticulated cranial element and many of the morphological features are difficult to observe or correctly interpret on articulated skulls. I expanded my comparisons beyond taxa that are closely related to *Uma* (other phrynosomatid lizards), taxa that are morphologically similar to phrynosomatid lizards (crotaphytid lizards), and taxa that are restricted to North America (North American teiids, scincomorphs, anguids, and other iguanian lizards). When possible, I examined more than one specimen from more than one locality as well as specimens of different sizes for each taxon, particularly for phrynsomatid taxa, in order to take account of intraspecific variation. I was generally not able to account for sexual dimorphism, because sex data were not available for most specimens. A list of comparative specimens is provided in Additional file 1.

### High-resolution computed tomography

Disarticulated skeletal specimens of *Uma exsul* and *Uma paraphygas* do not exist in museum collections and could not be made. I opted to scan the heads of alcohol-preserved specimens using high-resolution computed tomography to produce digital datasets of the skull for those species. I selected two relatively large adult specimens of both *Uma exsul* (TNHC 30247, 30248) and *Uma paraphygas* (TNHC 30594, 30596). All four specimens were scanned at the University of Texas at Austin High-Resolution X-Ray Computed Tomography Facility (UTCT) on an NSI scanner with a Fein Focus High Power source. The data sets include 1884 slices with a voxel size of 0.0136 mm for the *Uma exsul* scans, and 1795 slices with a voxel size of 0.0106 mm for the *Uma paraphygas* scans. The original slices were digitally resliced to provide three slice planes for data processing. I segmented the CT slices with the Avizo 9.7 Lite software using the magic wand tool with a minimum greyscale tolerance of 20,000-24,000. I used a lower minimum tolerance for relatively less dense bone, and occasionally used manual selections to segment bone. The images in Figs. 5e, f, 6e and f are surface renderings of the premaxilla in orthogonal view.

### Genetic data

#### Concatenated dataset

I obtained data for most species of *Uma* currently recognized by the Society for the Study of Amphibians and Reptiles [104] excluding the putative hybrid species *Uma rufopunctata* [33]. For outgroups, I included a representative taxon from each of the other sand-lizard genera and *Phrynosoma*, as well as the sceloporine *Urosaurus ornatus*. I used other iguanian outgroups to permit additional node calibrations, including a corytophanid (*Basiliscus basiliscus*), an agamid (*Leiolepis belliana*) and a chamaeleonid (*Chamaeleo calyptratus*). Most of the molecular data were downloaded from GenBank (GenBank accession numbers are in Additional file 2) and are primarily from Leaché et al. [67], Schulte and de Queiroz [79], and Townsend et al. [105]. Data for some nuclear loci are from Gottscho et al. 2017 [33] and were downloaded from DataDryad [106].

Sequences were aligned using the iterative refinement algorithm L-INS-i of MAFFT [107] implemented in AliView [108]. Nuclear protein-coding alignments were trimmed to start and end with the first and third codon positions and were checked for stop codons. I compiled the data with SequenceMatrix [109]. The dataset includes 37 nuclear protein-coding exons, six anonymous nuclear loci, and six mitochondrial genes, for a total of 40,215 total base pairs (bp). Eight of thirteen terminal taxa had 70% coverage (> 27,000 bp), but *Cophosaurus texanus* and *Holbrookia maculata* had 59.2 and 63.5% coverage respectively (25,536 and 23,823 bp), and *Uma inornata* had 22.7% coverage (9144 bp). Only mitochondrial genes were available for *Uma exsul* and *Uma paraphygas*, although that included all six genes and approximately 9.7% coverage (3900 bp) for each.

#### Coalescent dataset

I used a Sanger dataset of *Uma* from Gottscho et al. [32] and Gottscho et al. [33] of four protein-coding exons (BDNF, PNN, R35, and RAG-1). Those data were downloaded from DataDryad [106]. I included all operational taxonomic units (OTU) used by Gottscho et al. [33] and all specimens from the dataset, so the putative species *Uma* sp., *Uma cowlesi*, and the putative hybrid species *Uma rufopunctata* are OTUs in the coalescent analyses here. Individuals were assigned to OTUs following Gottscho et al. [33]. The dataset includes 31 specimens of *Uma scoparia*, 16 specimens of *Uma notata*, 18 specimens of *Uma inornata*, 6 specimens of *Uma rufopunctata*, 12 specimens of *Uma cowlesi*, and 5 specimens of *Uma* sp. I added *Callisaurus draconoides*, *Cophosaurus texanus*, and *Holbrookia maculata* as outgroups to allow the use of the sand lizard calibration, and those data were downloaded from GenBank (Additional file 2). I used single specimens per outgroup OTU for BDNF, PNN, and R35. However, the sequences identified on GenBank as RAG-1 for those outgroup specimens did not align with the RAG-1 data for the *Uma* specimens, so for *Callisaurus* and *Holbrookia* I arbitrarily selected data for the RAG-1 locus from another specimen in GenBank. The *Uma* specimens were already aligned, but I aligned the other sand lizards with the dataset with the L-INS-i algorithm in MAFFT.

### Non-clock phylogenetic analysis of concatenated dataset

I determined the best-fit partitioning scheme with PartitionFinder 2 [110] using a greedy search, the sample-size corrected Akaike information criterion, and the --raxml option [111]. The protein-coding nuclear loci were partitioned by codon position for the PartitionFinder analysis.

The partitioned concatenated data were analyzed using the Markov Chain Monte Carlo (MCMC) method in MrBayes 3.2.6 [112] for 1.5 × 10^7^ generations sampled every 1000 generations and for two separate independent runs. I used a GTR model and the Γ parameter for all partitions, and model parameters were unlinked across partitions. I conducted analyses on the Cyberinfrastructure for Phylogenetic Research (CIPRES) cluster [113]. Results were visualized in Tracer 1.6 to ascertain that the analysis reached stationarity and that effective sample size (ESS) values of > 200 were obtained for model parameters. Trees were summarized with the sumt command and the first 45% of samples were discarded as burn-in. The MrBayes block with all MrBayes analyses is in Additional file 3.

### Rationale and age distributions for node calibrations

Node calibrations were assigned following the best-practices suggested by Parham et al. [6]. I used four node calibrations for analyses of the concatenated dataset (Iguania, Acrodonta, Pleurodonta, and sand lizard node) and one calibration for analyses of the coalescent dataset (sand lizard node).

#### Iguania

The oldest fossil referred to Iguania is *Bharatagama rebbanensis* ([114]; specimens at University of Jammu, Geology Department collections, holotype VPL/JU/KR 66). Although some molecular analyses inferred an age for crown Iguania that postdates *Bharatagama* [105, 115] and one morphological analysis placed *Bharatagama* outside of Squamata [116], *Bharatagama* shares many apomorphies with acrodontans and is considered the earliest-known acrodontan [114, 117]. Hypotheses of squamate relationships may differ between morphological and molecular analyses [56, 118], but apomorphic characters of acrodontans are generally consistent regardless of which topology is preferred. Because it is unclear whether *Bharatagama* is a stem or crown acrodontan, I used *Bharatagama* to calibrate Iguania. Fossils of *Bharatagama* were recovered from the Upper Member of the Kota Formation, which was dated as Toarcian to ? Aelenian 183 ± 0.7 Ma – 170.3 ± 1.4 Ma [117, 119, 120]. I followed Benton et al. [117] in assigning the calibration a minimum age of 168.9 Ma and a soft maximum age of 209.5 Ma at the base of the Rhaetian (end Triassic).

I used an offset lognormal distribution to calibrate the age of the Iguania (root) node, using parameters similar to the analyses by Harrington and Reeder [121]. The minimum of the distribution was 168.9 Ma and the mean was 188.9 Ma. I used a standard deviation (SD) of 10.9 such that the upper 95% bound of the distribution was approximately 209.5 Ma.

#### Acrodonta

Cretaceous amber fossils from Myanmar exhibiting agamid apomorphies indicate the presence of Agamidae in the middle Cretaceous at ~ 99 Ma ([122]; Museum of Comparative Zoology, Harvard University, holotype MCZ R-190836). I used the holotype to calibrate the divergence of Agamidae from Chamaeleonidae. U-Pb dating of zircons preserved in the Burmese amber outcrops yielded a date of 98.79 ± 0.62 Ma [122, 123], so I assigned a minimum of 98.17 Ma to the Acrodonta node.

The designation of the Early Cretaceous fossil *Xianglong* as a crown acrodontan ([124]; Liaoning Paleontological Museum, Shenbei, Shenyang, China, holotype LPM 000666) is uncertain pending discovery of adult cranial material [125], so I used that fossil to set a soft maximum for crown Acrodonta. *Xianglong* was recovered from the Zhuanchengzi Bed of the Yixian Formation near Yizhou, Liaoning Province, China. ^40^Ar/^39^Ar data dated the Yixian Formation at 125.0 ± 0.18 [126].

I calibrated the Acrodonta node (the divergence between *Leiolepis belliana* and *Chamaeleo calyptratus*) with a lognormal distribution with a minimum age of 98.17 Ma, a mean of 110.7 Ma, and an SD of 8.2, such that the 95% bound of the distribution was approximately 124.82 Ma.

#### Pleurodonta

There is no consensus of relationships among the major clades of Pleurodonta (=Iguanoidea sensu Daza [127] and Iguanidae sensu Schulte et al. [128]) regardless of data type or analytical methodology [16, 39, 55, 56, 62, 69, 73, 115, 127–133]. Additionally, membership of some major clades differs between analyses and clade concepts (compare Polychrotidae sensu Conrad et al. [130] with Polychrotidae sensu Frost et al. [133]). The oldest iguanian fossils referred unambiguously using apomorphies to a pleurodontan clade with clear membership are stem corytophanid fossils of *Suzanniwana patriciana* from the earliest Eocene of Wyoming ([15]; University of California Museum of Paleontology, holotype UCMP 167664). I used those fossils to calibrate the node corresponding to the most recent common ancestor of Corytophanidae and Phrynosomatidae. The fossils were recovered from the lower Wildwood Formation of the Bighorn Basin at the locality UCMP V99019. The locality is within the carbon isotope excursion whose base denotes the Paleocene-Eocene boundary. The base of the carbon isotope excursion is at 56 Ma [134] and the entire excursion lasted ~ 170 ka [135], so I assigned a minimum age of 55.83 Ma to the Pleurodonta node.

Several Late Cretaceous fossils were assigned to or placed in Pleurodonta, including *Ctenomastax parva*, *Isodontosaurus gracilis*, *Polrussia mongoliensis*, *Saichangurvel davidsoni*, and *Temujinia ellisoni* [56, 127, 130, 136, 137]. Those fossils are not appropriate for calibrating the minimum age of Pleurodonta because none of the taxa are unambiguously referable to crown Pleurodonta [16, 121, 131, 138]. I used the fossils (which are roughly equal in age) to set a soft maximum age for crown Pleurodonta of 75 Ma, based on magnetostratigraphic dates for the Upper Cretaceous Ukhaa Tolgod locality in Mongolia [139] where *Saichangurvel davidsoni* ([136]; Institute of Geology, Mongolian Academy of Sciences, Ulaanbaatar, Mongolia, holotype IGM 3/858) was found.

I parametrized the Pleurodonta node (divergence between *Basiliscus basiliscus* and Phrynosomatidae) with an offset lognormal distribution with a minimum of 55.83 Ma and a mean of 62.83 Ma. The SD of the lognormal was 7 so that the 95% bound of the distribution was at approximately 75 Ma.

#### Sand lizard node

I assigned the *Uma* fossil LACM 159892 an age of 8.77 Ma (see above). The fossil is not referable to the crown clade of *Uma*, so I used the fossil to calibrate the divergence of *Uma* from the other sand lizards (*Callisaurus*, *Cophosaurus*, *Holbrookia*), as opposed to using it to calibrate the minimum age of the crown clade of *Uma*. Because of the uncertainty in the fossil record of the maximum age of the sand lizard clade (see below), I used the age of the crown sand lizard clade from a previous divergence time analysis, 26.55 Ma [39], to derive a mean for the calibration. I used an offset exponential calibration with an offset of 8.77 Ma and a mean of 25.65 Ma.

There are no fossils that predate the *Uma* fossil described here that were referred with apomorphies or by a phylogenetic analysis to the sand lizard clade that could be used to set a soft maximum age for the sand lizard node. Middle Miocene fossils described as having a suite of primitive sand lizard features were tentatively referred to *Holbrookia*? *antiqua* without an explicitly apomorphy-based diagnosis [27], so the fossils require further study. The earliest known phrynosomatid fossils are from the early Miocene Miller Local Fauna approximately 20–19 Ma ([11]). The fossils do not preserve any apomorphies allowing a more specific allocation than Phrynosomatidae. Some fossils older than those from the Miller local fauna were assigned to or allied with Phrynosomatidae, including the Eocene fossils of *Tuberculacerata personi* [14] and the Oligocene fossil *Phrynosoma* (*Paraphrynosoma*) *greeni* [140]. However, the purported phylogenetic affinities of those fossils with Phrynosomatidae were rescinded by the authors who described the fossils, and neither of those taxa is referable to the total clade Phrynosomatidae [5, 18].

### Strict-clock analysis of concatenated dataset

Uncalibrated strict-clock analyses were used to find informative clock rate priors for calibrated relaxed-clock analyses [141]. Strict-clock analyses were conducted in MrBayes v3.2.6 for 1.5 × 10^7^ generations sampled every 1000 generations for two independent runs. The tree topology in all strict-clock analyses was fixed to the topology found in the non-clock analysis. Strict-clock analyses were conducted using a base clock rate with a posterior distribution drawn from an exponential prior with a mean of 1 [141]. I estimated a single-clock rate prior for the entire dataset, and separate clock rates for the nuclear data and the mitochondrial data. I used those rates to parametrize models with a single clock rate and models with two clock rates.

### Relaxed-clock analysis of concatenated dataset

Calculations using the results of MrBayes analyses followed the procedures of Ronquist et al. [141]. I used the median TH from the MrBayes strict-clock analyses to calculate the mean clock rate for relaxed-clock analyses by dividing the median TH from each analysis by the mean age of the tree (188.9 Ma). I used a lognormal clock rate distribution, and the SD for the lognormal was calculated such that dividing the upper 95% estimate of the TH from the strict-clock analyses by the minimum age of Iguania (168.9 Ma) was 1 SD away from the mean of the lognormal. I determined clock rates of 1.175 × 10^− 3^ substitutions per site per million years (SSMY) for the one-rate model, 5.804 × 10^− 4^ SSMY for the nuclear data, and 8.557 × 10^− 3^ SSMY for the mitochondrial data. These values were similar to those used in divergence time analyses of *Uma* [32, 33] and of Hymenoptera [136]. I calibrated analyses with fossil calibrations (see above) at the Iguania, Pleurodonta, Acrodonta, Phrynosomatidae, and sand lizard nodes, which were all constrained to be monophyletic. In the analyses in which the western clade of *Uma* was constrained to have diverged in the Pleistocene, the age of the western *Uma* node was calibrated with a lognormal distribution with a mean of 0.95 Ma and an SD of 0.22, approximately the 95% HPD interval 1.34–0.60 Ma of Gottscho et al. [32].

Relaxed-clock analyses were conducted in BEAST v1.10 [74] because BEAST allows the use of more than one clock rate prior. Analyses were run with a Yule (pure-birth) model and an uncorrelated lognormal relaxed-clock. I used a GTR + Γ substitution model, unlinked across partitions, with empirical base frequencies. I ran each analysis for 8 × 10^7^ generations sampled every 1000 generations and conducted two independent runs for each model, and the first 20% of samples from each run were discarded as burn-in. The results of multiple BEAST runs were combined in LogCombiner v 1.10 and maximum clade credibility trees were extracted from the resulting tree files in TreeAnnotator v1.10. BEAST XML files are in Additional files 4, 5, 6, 7, 8, 9.

### Multispecies coalescent analysis

Multispecies coalescent analyses were performed with *BEAST in BEAST 2.5. I used the clock rates for each exon from Gottscho et al. [32], which were calculated from Townsend et al. [115]. Those rates are 2.2 × 10^− 3^ SSMY for BDNF, 2.19 × 10^− 3^ SSMY for RAG-1, 2.23 × 10^− 3^ SSMY for PNN, and 4.25 × 10^− 3^ SSMY for R35. A HKY + Γ substitution model was used for each locus and was unlinked across partitions, with empirical base frequencies. I used an uncorrelated lognormal relaxed-clock and a linear with constant root population size model. Monophyly of the western clade of *Uma* was constrained in all analyses. I performed analyses on three models, all of which contained the sand lizard fossil calibration. One model included only the fossil calibration, a second model contained a calibration in the Pliocene at the western *Uma* node that was derived from the results of the two-rate all fossil calibrations analysis (lognormal prior with a mean of 4.7 and an SD of 0.275), and a third model contained a Pleistocene calibration at the western *Uma* node that reflected the results of Gottscho et al. [32]. I ran two analyses for each model, which were all run for 2 × 10^8^ generations sampled every 10,000 generations. The first 10% of samples were discarded as burn-in. I created maximum clade credibility trees of the combined results of both runs of each model in TreeAnnotator 2.5. BEAST XML files are in Additional files 10, 11, 12.

### Model comparison

For the concatenated analyses, stepping stone and path-sampling analyses were run in BEAST v1.10 for 100 path steps and 8 × 10^5^ generations per step, for a total of 8 × 10^7^ generations. I conducted two separate runs for each model. The results of multiple runs were combined using code from Baele et al. [76] and Baele et al. [142] and marginal likelihoods were calculated in BEAST. For the coalescent analyses, stepping stone analyses were run in the PathSampler application in the BEAST 2 Model Selection package. Analyses were run for 100 path steps and 2 × 10^6^ generations per step for a total of 2 × 10^8^ generations, with a 50% burn-in and a 1 × 10^6^ generation pre-burn-in of the first step. Path-sampling and stepping-stone analyses outperform other Bayesian hypothesis testing and model selection methods [76, 77, 142].

## Additional files

Additional file 1: List of comparative specimens used in diagnosis of the fossil LACM 159892. (DOCX 89 kb)

Additional file 2: GenBank accession numbers for molecular data. Identifiers of sequences accessioned at DataDryad are also included. (XLSX 51 kb)

Additional file 3: Concatenated molecular data and MrBayes block for non-clock and strict-clock phylogenetic analysis. Additional file 3 also contains the maximum clade credibility tree files for the BEAST divergence time analyses with all fossil calibrations, as shown in Fig. 8. (TXT 547 kb)

Additional file 4: BEAST xml file for relaxed-clock analysis with one clock rate and all fossil calibrations. (XML 663 kb)

Additional file 5: BEAST xml file for relaxed-clock analysis with one clock rate and all fossil calibrations except for the sand lizard calibration. (XML 663 kb)

Additional file 6: BEAST xml file for relaxed-clock analysis with one clock rate, all fossil calibrations, and the western clade of *Uma* calibration in the Pleistocene. (XML 664 kb)

Additional file 7: BEAST xml file for relaxed-clock analysis with two clock rates and all fossil calibrations. (XML 667 kb)

Additional file 8: BEAST xml file for relaxed-clock analysis with two clock rates and all fossil calibrations except for the sand lizard calibration. (XML 667 kb)

Additional file 9: BEAST xml file for relaxed-clock analysis with two clock rates, all fossil calibrations, and the western clade of *Uma* calibration in the Pleistocene. (XML 667 kb)

Additional file 10: BEAST xml file for multispecies coalescent analysis with only the sand lizard calibration. (XML 311 kb)

Additional file 11: BEAST xml file for multispecies coalescent analysis with the sand calibration and the Pliocene calibration. (XML 312 kb)

Additional file 12: BEAST xml file for multispecies coalescent analysis with the sand calibration and the Pleistocene calibration. (XML 312 kb)

## Data Availability

The list of comparative specimens is available in Additional file 1. The high-resolution computed tomography data supporting the conclusions of this article are available at Morphosource https://www.morphosource.org/Detail/ProjectDetail/Show/project_id/704. The molecular data supporting the conclusions of this article are available at GenBank https://www.ncbi.nlm.nih.gov/genbank/ and at Dryad https://datadryad.org/resource/doi:10.5061/dryad.8br5c. Accession numbers for molecular data are in Additional file 2. The concatenated and coalescent molecular datasets and associated analysis parameters are available in Additional files 3, 4, 5, 6, 7, 8, 9, 10, 11, 12.

## Abbreviations

BF: Bayes factor
bp: Base pairs
CAS: California Academy of Sciences
CIPRES: Cyberinfrastructure for phylogenetic research
ESS: Effective sample size
HPD: Highest posterior density
IGM: Institute of Geology, Mongolian Academy of Sciences, Ulaanbaatar, Mongolia
LACM: Natural History Museum of Los Angeles County
LPM: Liaoning Paleontological Museum, Shenbei, Shenyang, China
MCMC: Markov Chain Monte Carlo
MCZ: Museum of Comparative Zoology, Harvard University
MSH: Mammoth Site Herpetological Comparative Collection
MVZ: Museum of Vertebrate Zoology, University of California at Berkeley
NALMA: North American Land Mammal Age
OTU: Operational taxonomic unit
SD: Standard deviation
SSMY: Substitutions per site per million years
TH: Tree height
TNHC: Texas Natural History Collections
TxVP: Texas Vertebrate Paleontology Collections
UCMP: University of California Museum of Paleontology
UTCT: University of Texas at Austin High-Resolution X-Ray Computed Tomography Facility
VPL/JU/KR: University of Jammu, Geology Department collections
WAM: Western Australian Museum
