# Supplementary material for: The first known fossil Uma: ecological evolution and the origins of North American fringe-toed lizards
Source: BMC Evol Biol. 2019 Sep 6;19:178. doi: 10.1186/s12862-019-1501-5 (PMC6729053; doi:10.1186/s12862-019-1501-5)
Supplement: Supplementary file 1 — List of comparative specimens used in diagnosis of the fossil LACM 159892. (DOCX 89 kb) [file 12862_2019_1501_MOESM1_ESM.docx]

**List of Comparative Specimens**

*Callisaurus draconoides*: TxVP M-8647, M-8648, M-8649, M-9130, M-9131, M-9132, M-9133, M-9134, M-12105, M-14320, M-14321; TNHC 95426

*Cophosaurus texanus*: TxVP M-8257, M-8650, M-9215, M-9217, M-9218, M-9219, M-9220, M-9221, M-9222, M-9223, M-9224, M-9225; M- 9226, M-9227; M-12104

*Holbrookia approximans* (*maculata*): TNHC 95319, 95320

*Holbrookia elegans*: TNHC 95322

*Holbrookia lacerata*: TNHC 95323, 95329, 95330, 95331, 95332, 95355, 95326

*Holbrookia maculata*: TxVP M-12128, M-14322; TNHC 18387, 35935, 95325, 95339, 95340, 95341

*Holbrookia propinqua*: TNHC 95326, 95327, 95328

*Petrosaurus mearnsi*: TxVP M-14323, M-14324, M-14910

*Petrosaurus thalassinus*: CAS 3009

*Phrynosoma coronatum*: TxVP M-9625

*Phrynosoma cornutum*: TxVP M-9621

*Phrynosoma hernandesi* (*douglassi*): TxVP M-9626

*Phrynosoma platyrhinos*: TxVP M-8646, M-8954

*Sceloporus clarkii*: TxVP M-12160

*Sceloporus graciosus*: TxVP M-12148

*Sceloporus jarrovi*: TxVP M-8485

*Sceloporus licki*: TxVP M9815

*Sceloporus magister*: TxVP M-12188

*Sceloporus occidentalis*: TxVP M-8917, M-8552

*Sceloporus orcutti*: TxVP M-12162

*Uma exsul*: MVZ 79603, 79605, 79606, 79607; TNHC 30247, 30248

*Uma inornata*: MVZ 58574, 58576, 77036

*Uma notata*: MVZ 78810, 78811; TxVP M-9950, M-14939; TNHC 100800

*Uma paraphygas*: TNHC 30594, 30596

*Uma scoparia*: CAS 42135; MVZ 39237; TxVP M-8533, M-8529, M-12119; TNHC 64522, 64523, 64524

*Urosaurus ornatus*: TxVP M-14329, M-14330

*Urosaurus graciosus*: TxVP M-14325, M-14326

*Urosaurus microscutatus*: TxVP M-14327, M-14328

*Uta stansburiana*: TxVP M-9954, M-14331, M-14332

*Crotaphytus bicinctores*: TxVP M-8612

*Crotaphytus collaris*: CAS 156251; TxVP M-12803

*Gambelia wislizenii*: CAS 200856; TxVP M-8394

*Basiliscus plumifrons*: TxVP M-9083, TxVP M-9085

*Basiliscus vittatus*: CAS uncatalogued; TxVP M-9086

*Laemanctus longpipes*: TxVP M-12779

*Ctenosaura* (*Enyaliosaurus*) *clarki*: TxVP M-9333

*Ctenosaura hemilopha*: TxVP M-9258

*Ctenosaura sp.*: TxVP M-9260, M-9261

*Dipsosaurus dorsalis*: TxVP M-9284, M-9285, M-9288

*Iguana iguana*: TxVP M- 8942, M-9451

*Sauromalus obesus*: TxVP M-8942, M-9781

*Chalarodon madagascariensis*: TxVP M-8509

*Oplurus cuvieri*: CAS 231484; TxVP M-8512

*Pristidactylus torquatus*: CAS 85234

*Leiocephalus cubensis*: CAS 39304

*Anolis sagrei* TxVP M-9043, M-9044

*Anolis trinitatis* TxVP M-9049

*Hoplocercus spinosus*: CAS 231483

*Morunasaurus groi*: CAS 98235

*Acanthosaura lepidogaster*: TxVP M-9797

*Agama agama*: TxVP M-8503

*Ctenophorus reticulatus*: WAM 167589

*Draco blanfordii*: TxVP M-9345

*Draco* sp: TxVP M-8652

*Hyrdrosaurus amboinensis*: MSH 1421

*Pogona minor*: WAM 112142, 162801

*Phrynocephalus theobaldi*: TxVP M-8457

*Uromastyx ornatus*: TxVP M-8438

*Chamaeleo calyptratus*: TxVP M-9145

*Chamaeleo dilepis*: TxVP M-8502

*Chamaeleo gracilis*: TxVP M-8634

*Furcifer pardalis*: TxVP M-8437

*Furcifer* sp.: TxVP M-8659

*Cordylus polyzonus*: TxVP M-8607

*Cordylus tropidosternum*: TxVP M-8602, M-8603, M-8495

*Cordylus warreni*: CAS 231486; TxVP M-8604, M-8605

*Gerrhosaurus flavigularis*: TxVP M-8610

*Gerrhosaurus major*: CAS 179086, 204767

*Gerrhosaurus validus*: TxVP M-8492

*Platysaurus* sp.: CAS 225589

*Zonosaurus laticaudatus*: TxVP M-8493, M-8611

*Zonosaurus madagascariensis*: TxVP M-8494

*Xantusia henshawi*: TxVP M-8571, M-8572

*Plestiodon inexpectatus*: CAS 179090

*Plestiodon obsoletus*: CAS 71603

*Anguis fragilis*: CAS 55193

*Diploglossus pleei*: CAS 200840

*Elgaria multicarinata*: TxVP M-8988

*Mesaspis moreletti*: TxVP M-8579

*Ophiodes striatus*: CAS 231485

*Ophisaurus ventralis*: CAS 74296

*Pseudopus* (*Ophisaurus*) *apodus*: TxVP M-9002

*Xenosaurus grandis*: TxVP M-8960

*Coleonyx variegatus*: TxVP M- 8520, TxVP-8521

*Hemidactylus turcicus*: TxVP M-8523
